# Identification of a distinct cluster of GDF15^high^ macrophages induced by *in vitro* differentiation exhibiting anti-inflammatory activities

**DOI:** 10.3389/fimmu.2024.1309739

**Published:** 2024-04-08

**Authors:** Chaochao Dai, Hongyu Zhang, Zhijian Zheng, Chun Guang Li, Mingyuan Ma, Haiqing Gao, Qunye Zhang, Fan Jiang, Xiaopei Cui

**Affiliations:** ^1^Key Laboratory of Cardiovascular Proteomics of Shandong Province and Department of Geriatric Medicine, Qilu Hospital of Shandong University, Jinan, Shandong, China; ^2^Key Laboratory of Cardiovascular Remodeling and Function Research (Chinese Ministry of Education and Chinese National Health Commission), Qilu Hospital of Shandong University, Jinan, Shandong, China; ^3^NICM Health Research Institute, Western Sydney University, Westmead, NSW, Australia

**Keywords:** growth differentiation factor 15, GDF15, macrophage, anti-inflammatory, single-cell RNA sequencing, cell population, human

## Abstract

**Introduction:**

Macrophage-mediated inflammatory response may have crucial roles in the pathogenesis of a variety of human diseases. Growth differentiation factor 15 (GDF15) is a cytokine of the transforming growth factor-β superfamily, with potential anti-inflammatory activities. Previous studies observed in human lungs some macrophages which expressed a high level of GDF15.

**Methods:**

In the present study, we employed multiple techniques, including immunofluorescence, flow cytometry, and single-cell RNA sequencing, in order to further clarify the identity of such GDF15^high^ macrophages.

**Results:**

We demonstrated that macrophages derived from human peripheral blood mononuclear cells and rat bone marrow mononuclear cells by *in vitro* differentiation with granulocyte-macrophage colony stimulating factor contained a minor population (~1%) of GDF15^high^ cells. GDF15^high^ macrophages did not exhibit a typical M1 or M2 phenotype, but had a unique molecular signature as revealed by single-cell RNA sequencing. Functionally, the *in vitro* derived GDF15^high^ macrophages were associated with reduced responsiveness to pro-inflammatory activation; furthermore, these GDF15^high^ macrophages could inhibit the pro-inflammatory functions of other macrophages via a paracrine mechanism. We further confirmed that GDF15 *per se* was a key mediator of the anti-inflammatory effects of GDF15^high^ macrophage. Also, we provided evidence showing that GDF15^high^ macrophages were present in other macrophage-residing human tissues in addition to the lungs. Further scRNA-seq analysis in rat lung macrophages confirmed the presence of a GDF15^high^ sub-population. However, these data indicated that GDF15^high^ macrophages in the body were not a uniform population based on their molecular signatures. More importantly, as compared to the *in vitro* derived GDF15^high^ macrophage, whether the tissue resident GDF15^high^ counterpart is also associated with anti-inflammatory functions remains to be determined. We cannot exclude the possibility that the *in vitro* priming/induction protocol used in our study has a determinant role in inducing the anti-inflammatory phenotype in the resulting GDF15^high^ macrophage cells.

**Conclusion:**

In summary, our results suggest that the GDF15^high^ macrophage cells obtained by *in vitro* induction may represent a distinct cluster with intrinsic anti-inflammatory functions. The (patho)physiological importance of these cells *in vivo* warrants further investigation.

## 1 Introduction

Macrophages mediate innate immunity under homeostasis and play essential roles in regulating tissue inflammation under pathological conditions. Macrophages display remarkable heterogeneity and plasticity, and can change their physiology in response to environmental cues, giving rise to different populations with distinct functions in different organs (1–5). It is well established that in the body, macrophages originate from two major sources, namely embryonic hematopoietic precursors located in embryonic hematopoietic organs (such as yolk sac and fetal liver), and definitive hematopoietic stem cells located in the bone marrow (4, 6). Most tissue-resident macrophages are derived from the embryonic origins, although to varying degrees circulating monocytes have a contribution in replenishing the pool during postnatal life. During inflammation, in contrast, the majority of macrophages infiltrating the inflamed tissue arise from circulating monocytes (4, 6). In the lungs, for example, there are two major macrophage populations, namely alveolar macrophage and interstitial macrophage (7, 8). With inflammatory insults, additional monocytes/macrophages are recruited from the circulation and may expand locally (8). Macrophage-mediated inflammation is implicated in the pathogenesis of a variety of lung diseases, including pulmonary arterial hypertension (PAH) (7), chronic obstructive pulmonary disease (COPD) (9), inflammatory acute lung injury (10), and pulmonary fibrosis (11).

Growth differentiation factor 15 (GDF15) is a cytokine of the transforming growth factor-β (TGF-β) superfamily. It was initially identified as a macrophage-derived cytokine which had inhibitory effects on lipopolysaccharide (LPS)-induced macrophage activation (thus also known as macrophage inhibitory cytokine 1/MIC-1) (12). In addition to macrophages, GDF15 is also highly expressed in the epithelial cells in different organs (13). Accumulating evidence from *in vivo* functional studies has suggested that GDF15 may be an important regulator of inflammation in the body; for example, GDF15 has been shown to have inhibitory effects on experimental systemic lupus erythematosus (14) and glomerulonephritis (15). Moreover, GDF15 exhibits protective effects in several inflammatory metabolic disorders (*e.g.* non-alcoholic fatty liver disease and type 2 diabetes mellitus) (16) (17) and cardiovascular diseases (*e.g.* myocardial infarction and atherosclerosis) (18–20). Traditionally, monocyte-derived macrophages are classified into M1 (pro-inflammatory) and M2 (anti-inflammatory and regenerative) phenotypes. It has been observed that GDF15 may facilitate the differentiation of macrophages toward the anti-inflammatory M2 phenotype (20, 21). In previous studies, researchers identified some macrophage cells in human lung tissues from both healthy subjects and patients with PAH, which exhibited a high level of GDF15 expression (22, 23). We termed these cells as GDF15^high^ macrophages thereafter in this article. GDF15^high^ macrophages were found in the alveoli; however, in PAH lungs GDF15^high^ macrophages were also present in the peri-arterial area (23). The functional properties of the GDF15^high^ macrophage remain unknown.

Nowadays, the cutting-edge single-cell RNA sequencing (scRNA-seq) technology empowers people to determine cell heterogeneities based on the genomic signature at a single cell level, and the emerging evidence has suggested that the macrophage heterogeneity in a single tissue is much more complex than expected (24). Recently, Patsalos and colleagues discovered a distinct population of macrophages with tissue regenerative functions (which were called “repair monocyte-derived macrophages”) in a murine model of sterile inflammatory muscle injury (25). Interestingly, these researchers demonstrated that GDF15 was not only a marker, but also a central mediator of the repair-type macrophages, which acted in local, autocrine, as well as paracrine manners to promote sustained regenerative transcriptional programs (25). However, the relevance of the GDF15^high^ population of macrophages in human is to be established. In the present study, therefore, we aim to clarify whether GDF15^high^ macrophages represent a functionally distinct population which has anti-inflammatory functions. We have provided evidence showing that (1) macrophages derived from human peripheral blood mononuclear cells (PBMNCs) by *in vitro* differentiation contain a minor population (~1%) of GDF15^high^ cells; (2) scRNA-seq confirmed that GDF15^high^ macrophages represent a distinct minor population with a unique molecular signature; (3) the *in vitro* derived GDF15^high^ macrophage is associated with intrinsic anti-inflammatory functions.

## 2 Materials and methods

### 2.1 Materials

Recombinant GDF15 (#10596-GD) and recombinant interferon (IFN)-γ (#585-IF) were purchased from R&D Systems (Minneapolis, MN, USA). Recombinant human granulocyte-macrophage colony stimulating factor (GM-CSF) and rat GM-CSF were from Novoprotein Technology (#C003 and #CB91 respectively) (Suzhou, Jiangsu Province, China). Monocrotaline and lipopolysaccharides (LPS) was purchased from Merck KGaA (Darmstadt, Germany).

### 2.2 Human and animal ethics

Experiments involving human samples were approved by the Human Ethics Committee of Shandong University Cheeloo College of Medicine (document #KYLL-201810-054). Informed consents were obtained before commencement of the study. Experiments involving animals were approved by the Institutional Animal Ethics Committee of Shandong University Cheeloo College of Medicine (document #KYLL-2018ZM-636) and conducted in accordance with the Animals in Research: Reporting *In Vivo* Experiments (ARRIVE) guidelines. Animals were handled in accordance with the National Institutes of Health guide for the care and use of laboratory animals (NIH Publications No. 8023, revised 1978).

### 2.3 Human tissue specimens

Human lung and kidney tissues were obtained from patients undergoing surgical tumor resections. The non-cancerous part of the tissue block was dissected and preserved for the present study. Some of the lung tissues were complicated with COPD; those without COPD were treated as normal lung tissues. Atherosclerotic arterial intima specimens were obtained from patients undergoing carotid endarterectomy. Colon tissues were either biopsies from ulcerative colitis patients undergoing colonoscopy or donations from healthy subjects undergoing routine health checks with colonoscopy. Tissue blocks were fixed in 4% paraformaldehyde solution for 24 hr at room temperature, and embedded in paraffin. Sections of 5 μm in thickness were cut.

### 2.4 Isolation and differentiation of human PBMNCs

Seven healthy volunteers and 6 PAH patients were recruited in our study. Their basic demographic information was given in **Supplementary Table S1**. From each subject, 20 mL of peripheral venous blood was withdrawn into a heparinized tube, and diluted 1:1 in sterile PBS. PBMNCs were purified using a Ficoll-based reagent kit from TBD Science (#LTS1077, Tianjin, China). Cells (10^6^ per mL) were cultured and differentiated in RPMI 1640 medium containing 10% fetal bovine serum (all from Thermo Fisher Scientific, Waltham, MA, USA) plus 20 ng/mL of human GM-CSF as described (26). At day 3, half of the culture medium was replaced with fresh GM-CSF-containing medium. At day 7, adherent macrophages were collected for further experimentations.

### 2.5 CD68 promoter driven-GFP transgenic reporter (CD68pro-GFP) rats

A transgenic reporter rat strain (on Sprague Dawley background) expressing enhanced GFP selectively in monocytes/macrophages was created using the strategy described previously (27). Briefly, the transgene construct was prepared by inserting a human CD68 promoter upstream of the GFP open reading frame. This strategy allowed stable GFP expression in both blood monocytes and mature tissue resident macrophages (27). The cloning service and breeding pair establishment were provided by Cyagen Biosciences (Suzhou, Jiangsu Province, China). Rats were housed in standard open-top polycarbonate cages with corn cob bedding, and maintained in an air-conditioned environment (non-SPF) with 12-hour light/dark cycles. Standard laboratory rodent diet and autoclaved tap water were provided *ad libitum*.

### 2.6 Isolation and differentiation of rat bone marrow mononuclear cells (BMMNCs)

Male CD68pro-GFP rats (six weeks of age, body weight 110-120 g) were used for primary BMMNC isolation. Rats were euthanized by intraperitoneal injection of an overdose of sodium pentobarbital. BMMNCs were harvested from the femur and tibia as described previously (28). Cells were resuspended in Dulbecco’s modified Eagle medium (from Thermo Fisher) to a density of 10^6^ per mL, and cultured in the presence of 10% fetal bovine serum. Macrophage differentiation was induced by treating with rat GM-CSF at 20 ng/mL for 7 days.

### 2.7 Co-culture experiments

RAW264.7 murine macrophages were cultured in Dulbecco’s modified Eagle medium with 10% fetal bovine serum as described previously (29). For co-culture experiments, 6-well transwell plates (0.4 μm pore size) (from Corning, New York, USA) were used. Differentiated rat macrophages were fractionated by Fluorescence-activated cell sorting (FACS). 2×10^4^ fractionated cells were resuspended in 1 mL of Dulbecco’s modified Eagle medium with 10% fetal bovine serum and seeded in the upper chamber of each well. 2×10^5^ RAW264.7 cells in the same medium were seeded in each lower chamber. Cells were co-cultured for 48 hr, then the RAW264.7 cells were either left untreated or treated with LPS (1 μg/mL) for 6 hr.

### 2.8 Rat PAH models and lung macrophage isolation

Monocrotaline-induced PAH in CD68pro-GFP rats was established by a single bolus injection (50 mg/kg subcutaneously) as described previously (30). Alveolar macrophages were isolated by bronchoalveolar lavage (31). Rats were euthanized; the trachea was exposed and intubated. The lungs were flushed with cold PBS buffer containing 0.6 mM EDTA (10 mL × 3 times). Cells in the lavage fluid were collected by centrifugation (400 × g for 10 min at 4°C). Interstitial macrophages were isolated by enzymatic digestion (32). The lungs were perfused with the PBS-EDTA buffer via the right ventricle until the color was pale. The peripheral lung tissue was cut into small pieces, and incubated in a digestion buffer containing 50 mM TES (pH 7.4), 0.36 mM CaCl_2_, 0.01% DNase I, 10% fetal bovine serum, and 1 mg/mL collagenase type I, for 60 min at 37°C. The tissues were dissociated by aspiration, and the mixture was passed through a 30-μm nylon mesh. The cells were collected by centrifugation, washed twice with PBS, and resuspended in RPMI-1640 medium containing 15% fetal bovine serum. The cells were transferred to a culture dish, and cultured for 2 hr at 37°C in an CO_2_ incubator. The floating cells and debris were removed by gently washing the culture dish with warm medium. The adherent interstitial macrophage cells were detached by trypsin treatment and pooled with the alveolar macrophage fraction.

### 2.9 scRNA-seq and data processing

*In vitro* differentiated human macrophages were detached with 0.25% trypsin-EDTA solution and single cell suspension was obtained by filtering through a 70-µm cell strainer. Fresh lung alveolar and interstitial macrophages were isolated from rats as described above. Cell viability was confirmed by staining with 0.2% trypan blue solution in PBS (pH 7.2). Library construction and double-end sequencing services were provided by BGI (Shenzhen, Guangdong Province, China), using Chromium Single Cell 3’ Reagent Kits v2 (from 10x Genomics, Pleasanton, CA, USA) and NovaSeq 6000 System (Illumina, San Diego, CA, USA) respectively. The scRNA-seq raw data were imported into Cell Ranger Single-Cell Software Suite (version 4.0, 10x Genomics) and mapped to the GRCh38 human reference genome to generate digital gene expression matrices. Subsequently, the data set was processed with Seurat R package (version 4.03), including data cleaning, doublet removal, normalization, identification of highly variable (top 2000) genes, data integration (using Canonical Correlation Analysis), and dimensionality reduction (using Principal Component Analysis) (33). The “Find-Neighbors” and “Find-Clusters” modules in Seurat were used for establishment of cell sub-populations, and the results were visualized using the “Run-UMAP” function. Specific marker genes were identified using the “Find-All-Markers” function with Wilcoxon rank sum test.

### 2.10 Bioinformatics analysis

Differentially expressed genes (DEGs) were selected using cutoff values of fold change 1.5 and adjusted *P* 0.05 (Wilcoxon Rank-Sum test). Functional annotation and enrichment analysis for gene lists were carried out using DAVID platform (version 2021) (34). Cell-cell communication networks were constructed using CellChat software (35).

### 2.11 Immunofluorescence

Anti-CD68 (#66231-2-Ig from Proteintech, Wuhan, Hubei Province, China) and anti-GDF15 (#A0185 from ABclonal, Wuhan) were used for immunofluorescence staining. Tissue sections were deparaffinized and heated in 10 mM sodium citrate buffer (pH 6.0) in a microwave oven for 15 min for antigen retrieval. Tissues were permeabilized by incubating in 0.1% Triton X-100 solution at room temperature for 10 min, and blocked in 1% bovine serum albumin solution at room temperature for 30 min. Primary antibody treatment was carried out at 4°C for overnight, followed by fluorophore-conjugated secondary antibody treatment at room temperature for 60 min. For cellular experiments, cells were cultured on glass slides. Specimens were fixed in 4% paraformaldehyde for 10 min. Other steps were the same as described above. The nuclei were counterstained with DAPI for 1 min. Fluorescent images were taken using a fluorescence microscope (Model BX43, OLYMPUS, Tokyo, Japan) and analyzed using Image-J software (NIH). For each slide, 3 to 5 random fields were selected for analysis. Morphological analysis was carried out in a blind manner.

### 2.12 Flow cytometry and FACS

The following antibodies were used: anti-CD68 (#66231-2-Ig), anti-CD86 (#APC-65165), anti-CD80 (#PE-65083), anti-CD206 (#APC-65155) and anti-CD163 (#APC-65169) were from Proteintech; anti-GDF15 (#66231-2-Ig) was from ABclonal; anti-GDF15 (#BM5690) was from Boster (Wuhan); anti-TNFSF9 (tumor necrosis factor superfamily member 9) (#bs-3851R-APC) was from Bioss (Beijing, China); anti-interleukin (IL)-1β (#12-7018-41) was from Thermo Fisher; anti-IL-4 (#500808) was from BioLegend (San Diego, CA, USA). Cells detached from culture plates were filtered through 70-μm cell strainers. Rat cells were blocked with purified mouse anti-rat FcγII receptor (#550270 from BD Biosciences, Franklin Lakes, NJ, USA). Human cells were blocked by Human TruStain FcX Fc receptor blocking solution (#422301 from BioLegend). Flow cytometry analysis was performed using an AccuriC6 Plus platform; FACS was performed using a FACSAria III cell sorter (both from BD Biosciences). Data analysis and plotting were performed using FlowJo software (Version 10) (from BD). For 2-color labeling experiments, PE- and APC-conjugated antibodies were used in combination. compensation for the fluorescent spectral overlap was set up according to the instrument user’s manual.

### 2.13 Quantitative real-time PCR (qPCR)

Total RNA was extracted using TRIzol Reagent (Thermo Fisher) and the concentration was determined using NanoDrop 2000 spectrophotometer (Thermo Fisher). cDNA was synthesized using PrimeScript RT-PCR Kit from TaKaRa (#RR037A) (Otsu, Shiga, Japan). The PCR reaction was carried out using SYBR green qPCR mix (#CW0957M, from CoWin Biosciences, Taizhou, Jiangsu Province, China) on StepOne Real-Time PCR System (Thermo Fisher). β-actin was used as the house-keeping gene. The relative level of mRNA expression was calculated using the 2^-ΔΔCt^ method. Sequences of the primers used were given in **Supplementary Table S2**.

### 2.14 ELISA assay

Measurements of the protein levels of TNFSF9, GDF15 and annexin A1 were performed using ELISA kits from JYM-BIO (Wuhan). Standard curves were constructed according to the manuals; all r^2^ values of the standard curves were > 0.98.

### 2.15 Data and statistics

Data were expressed as mean ± standard error of the mean (SEM). Statistical analysis was performed using GraphPad Prism (version 8.0) (from GraphPad, San Diego, CA, USA). Unpaired *t*-test was used to compare two groups; one-way analysis of variance (ANOVA) followed by *post hoc* Tukey’s test was used to compare multiple groups. All tests were run as two-tailed. A value of *P <* 0.05 was considered as significant. The *n* number shown represented independent experiments/specimens, not technical replicates.

## 3 Results

### 3.1 Existence of GDF15^high^ macrophages in human lung tissues

**Figure 1:**
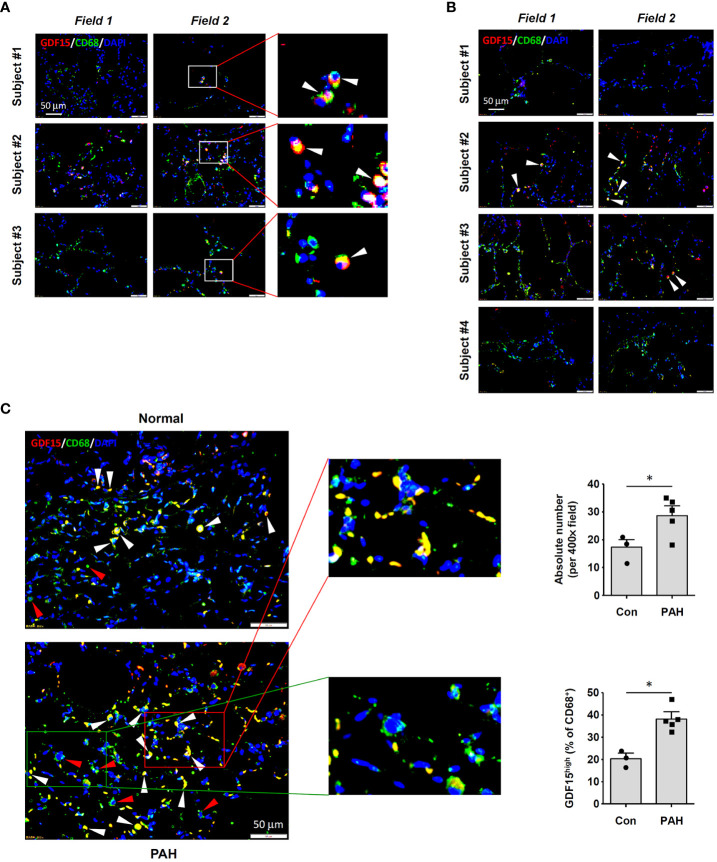
Immunofluorescence double labeling showing the existence of GDF15^high^ macrophages in lung tissues. **(A)** Results obtained in healthy human lung tissues from 3 independent subjects. Two representative microscopic fields were shown. Arrowheads indicated the CD68^+^GDF15^high^ macrophages. **(B)** Results obtained in human lung tissues with COPD from 4 independent subjects. Arrowheads indicated the CD68^+^GDF15^high^ macrophages. **(C)** Results obtained in rat lung tissues without and with experimental PAH. The nuclei were counterstained with DAPI (blue). White arrowheads indicated CD68^+^GDF15^high^ macrophages. The red box highlighted the presence of CD68^+^GDF15^high^ macrophages (white arrowheads); the green box highlighted the presence of CD68^+^GDF15^low^ macrophages (red arrowheads). The bar graphs showed the absolute and relative abundances of CD68^+^GDF15^high^ macrophages in normal and PAH lungs. Data were expressed as mean ± SEM. * *P <* 0.05, unpaired *t*-test.

To precisely confirm the existence of GDF15^high^ macrophages *in vivo*, we performed immunofluorescence double labeling for CD68 and GDF15 in human lung tissues without and with COPD respectively. A small population of CD68^+^ cells with a high level of GDF15 expression could be observed in normal lung tissues (**Figure 1A**). In **Figure 1B**, we showed that GDF15^high^ macrophages were also present in COPD lungs. We counted the frequencies of GDF15^high^ macrophages, demonstrating that these cells accounted for around 10-30% of the total CD68^+^ macrophages in both normal and COPD lungs (average 20.2% in normal and 22.2% in COPD lungs) (**Supplementary Table S3**). The limited number of samples prevented us from quantitatively comparing the absolute abundances of GDF15^high^ macrophages between normal and COPD lungs. However, given the comparable relative proportions of GDF15^high^ macrophages in normal and COPD lungs, and the marked increase in the total number of infiltrating macrophage cells in COPD lungs as reported previously (36), we argued that the actual abundance of GDF15^high^ macrophages in COPD lungs was likely to be increased. We noted that the abundances of GDF15^high^ macrophages in COPD Subjects #1 and #4 were sparse as compared to Subjects #2 and #3 (see **Figure 1B**). Therefore, we performed conventional histopathology examinations. As shown in **Supplementary Figure S1**, Subjects #1 and #4 had severe emphysema, resulting in poor cellularity of the lung tissue; in comparison, the lungs of Subjects #2 and #3 had clusters of dense inflammatory infiltrations. Supporting our argument above, it was suggested that the actual abundance of GDF15^high^ macrophages in COPD lungs was proportional to the total number of infiltrating macrophages.

### 3.2 Existence of GDF15^high^ macrophages in rat lung tissues

Consistent with the findings in human tissues, we demonstrated that GDF15^high^ macrophages were also present in rat lung tissues (**Figure 1C**). The abundance of GDF15^high^ macrophages was significantly increased in the lungs from PAH models (**Figure 1C**). This finding was consistent with previous reports showing that the abundance of GDF15^high^ macrophages was significantly increased in the lungs from patients with systemic sclerosis-associated PAH (22). Moreover, we observed that some of the GDF15^high^ macrophages in rat lungs (especially in PAH) were located in the interstitial area, suggesting that these might be interstitial macrophages.

### 3.3 GDF15^high^ macrophages can be differentiated from PBMNCs and BMMNCs

**Figure 2:**
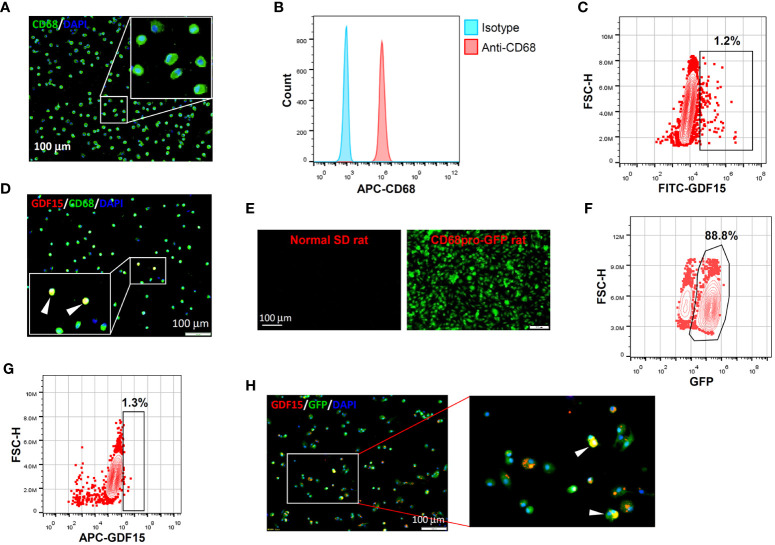
GDF15^high^ macrophages could be derived by *in vitro* differentiation of mononuclear cells. **(A, B)** Immunofluorescence staining and flow cytometry results confirming that *in vitro* differentiation of human peripheral blood mononuclear cells (PBMNCs) with GM-CSF for 7 days yielded CD68^+^ macrophages. **(C, D)** Flow cytometry and immunofluorescence double labeling results showing that the PBMNC-derived macrophages contained a minor population of GDF15^high^ cells (arrowheads in **D**) (example from 3 independent experiments). **(E, F)** Fluorescence microscopy and flow cytometry data showing that GM-CSF differentiation of rat bone marrow mononuclear cells (BMMNCs) *in vitro* yielded macrophages (GFP expressing) of a high purity (~90%). CD68pro-GFP rats had a GFP transgene under the control of CD68 promoter. Cells from normal rats showing no GFP fluorescence served as a negative control (left panel in **E**). **(G, H)** Flow cytometry and immunofluorescence double labeling data (from 3 independent experiments) showing that the BMMNC-derived macrophages (from CD68pro-GFP rats) contained a minor population of GDF15^high^ cells (arrowheads in **H**). The flow cytometry data in panels **(C, G)** were from cells gated for GFP^+^. The nuclei were counterstained with DAPI (blue).

*In vitro* differentiation of human PBMNCs with GM-CSF yielded a population of CD68^+^ macrophages confirmed by both immunofluorescence and flow cytometry (**Figures 2A, B**). Within these induced macrophages, around 1% of them exhibited a high level of GDF15 expression as determined by flow cytometry (**Figure 2C**). We further confirmed the existence of such GDF15^high^ macrophages using immunofluorescence (**Figure 2D**). Similar results were also obtained in rat cells. Using CD68pro-GFP rats, we showed that ~90% of GM-CSF-differentiated BMMNCs were macrophages (*i.e.* GFP expressing) (**Figures 2E, F**). As a negative control, macrophages derived from normal non-transgenic rats showed no GFP fluorescence (**Figure 2E**). Consistently, we demonstrated that around 1% of the BMMNC-derived macrophages were GDF15^high^ (**Figures 2G, H**).

### 3.4 *In vitro* derived GDF15^high^ macrophages do not exhibit a typical M1 or M2 phenotype

**Figure 3:**
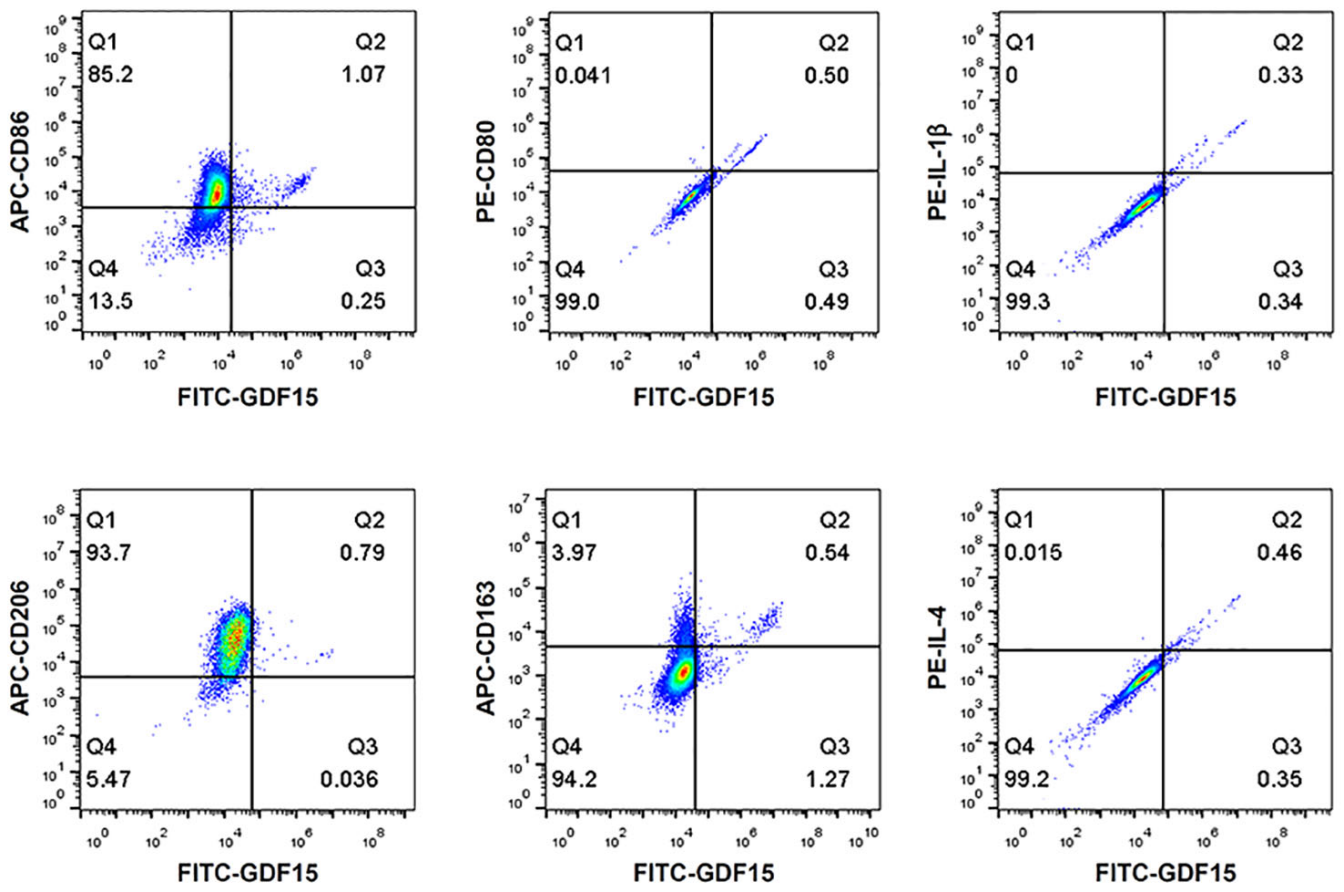
Flow cytometry results showing that GDF15^high^ macrophages did not exhibit a typical M1 or M2 phenotype. Experiments were performed in human PBMNC-derived macrophages, using CD86, CD80 and IL-1β as the M1 markers, and CD206, CD163 and IL-4 as the M2 markers. Data were from a single test using pooled samples from 4 healthy volunteers.

To understand whether the GDF15^high^ macrophages belonged to the conventional M1 or M2 phenotype, we performed flow cytometry characterization in human PBMNC-derived macrophages. We tested the M1 markers CD86, CD80 and IL-1β, and the M2 markers CD206, CD163 and IL-4. As shown in **Figure 3**, GDF15^high^ macrophages were primarily CD206^+^. CD163 and CD86 were only expressed in part of the GDF15^high^ macrophages (*i.e.* CD163^+/-^ and CD86^+/-^). However, we found that there were nice correlations between the expression levels of GDF15 and those of CD80, IL-1β and IL-4 (**Figure 3**). Taken together, these data indicate that the GDF15^high^ population belongs to neither the conventional M1 phenotype nor the M2 phenotype.

### 3.5 Molecular characterization of *in vitro* derived human GDF15^high^ macrophages using scRNA-seq

**Figure 4:**
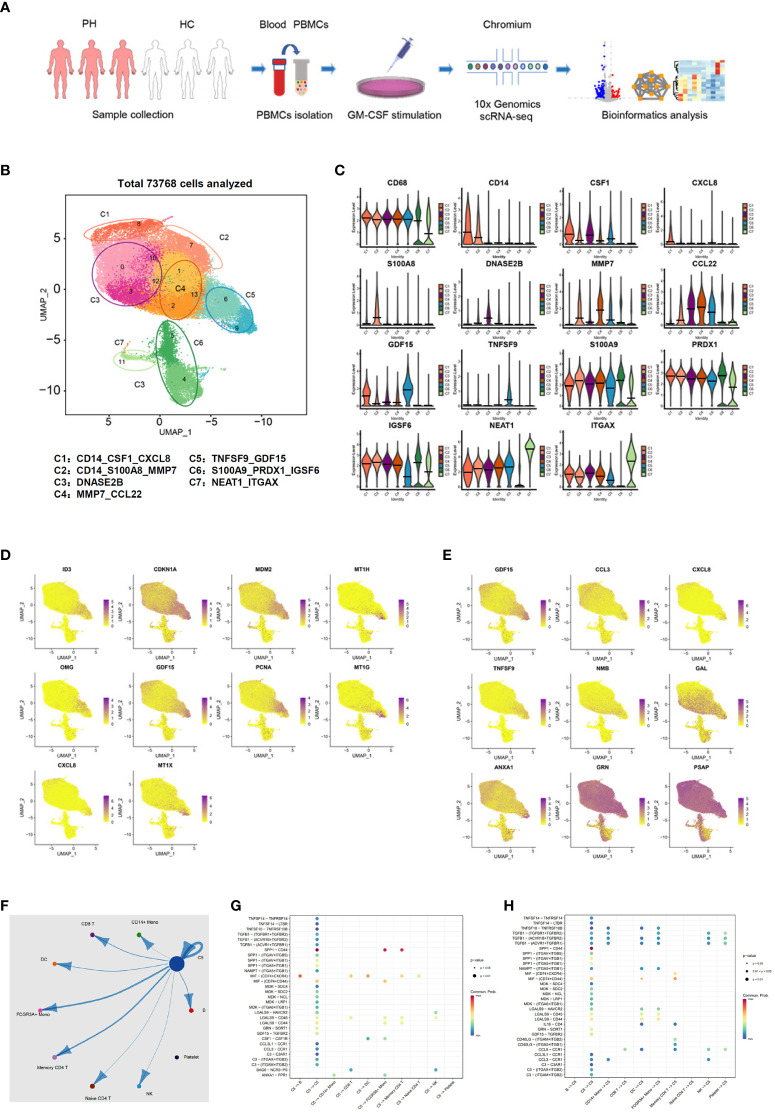
Molecular characterization of human PBMNC-derived GDF15^high^ macrophages with scRNA-seq. **(A)** Graphical outline of the experimental procedure. **(B)** UMAP plots showing the identified cell sub-populations (C1 to C7) based on the scRNA-seq data from total 73,768 cells pooled from samples of 3 healthy volunteers, 3 PAH patients harboring mutations in BMPR2 gene, and 3 PAH patients without BMPR2 mutations. The putative nomenclatures for C1 to C7 were given below the graph. The numbers 0 to 13 demarcated the initial cell clusters obtained with the default clustering process of Seurat. **(C)** Violin plots showing expression patterns of the identified marker genes for C1 to C7. The horizontal bars represented median values. **(D)** UMAP plots showing expression patterns of the top 10 genes that were overexpressed in GDF15^high^ macrophages (C5) as compared to GDF15^low^ cells. **(E)** UMAP plots showing expression patterns of the top 9 genes encoding secreted proteins which were overexpressed in GDF15^high^ macrophages as compared to GDF15^low^ cells. **(F)** Cell-cell communication network map created using CellChat showing the possible effector cells of the GDF15^high^ macrophage. **(G, H)** Predicted ligand-receptor pairs potentially involved in the signaling of reciprocal communications between GDF15^high^ macrophage and other cell types as listed in **(F)**.

To further confirm the existence of GDF15^high^ macrophages and delineate the molecular characteristics of these cells, we applied scRNA-seq in human PBMNC-derived macrophages. We obtained PBMNC-derived macrophages from 3 healthy volunteers (6958, 6946 and 5430 cells from each subjects respectively), 3 PAH patients harboring mutations in bone morphogenetic protein receptor type II (BMPR2) (7178, 13748 and 9739 cells respectively), and 3 PAH patients without BMPR2 mutations (6840, 8529 and 8400 cells respectively). Combined analysis of all 9 samples was performed using Seurat, and global uniform manifold approximation and projection (UMAP) plots were created (**Figure 4A**). Initial 14 cell clusters (numbers 0 to 13 in **Figure 4B**) were obtained with the default clustering process in Seurat; all of these clusters expressed the macrophage marker CD68. We observed that the cells in some different clusters expressed similar signature genes. In this case, these clusters were merged together as a single sub-population. As a result, 7 distinct macrophage sub-populations were established and the following putative nomenclatures were assigned (see **Figure 4B**): CD14_CSF1_CXCL8 (including cluster 8); CD14_S100A8_MMP7 (including cluster 7); DNASE2B (including clusters 0, 3, 10 and 12); MMP7_CCL22 (including clusters 1, 2 and 13); TNFSF9_ GDF15 (including clusters 6 and 9); S100A9_PRDX1_IGSF6 (including clusters 4 and 5); NEAT1_ITGAX (including cluster 11). Separate analysis and comparison of the data between healthy controls and PAH patients showed virtually identical clustering profiles of the macrophages (**Supplementary Figure S2**). Moreover, quantitative analysis of the relative cell abundance in each of these sub-populations demonstrated that there were no significant differences between healthy subjects and PAH patients with or without BMPR2 mutations (**Supplementary Figure S3**). The expression patterns of the identified marker genes in different sub-populations were shown in **Figure 4C** and **Supplementary Figure S4**, confirming that these macrophage sub-populations possessed distinct transcriptional signatures. Of note, our scRNA-seq data confirmed the existence of a sub-population of macrophages which exhibited a relatively high level of GDF15 expression (sub-population C5 in **Figure 4B**).

Next, we searched for DEGs between GDF15^high^ and GDF15^low^ macrophages and performed functional clustering analysis. In the healthy control subjects, there were 81 upregulated genes and 58 downregulated genes in the GDF15^high^ macrophages as compared to GDF15^low^ macrophages (**Supplementary Figure S5**, **Supplementary Table S4**). The total number of DEGs in cells from PAH patients was less than that of healthy subjects; however, these regulated genes were highly consistent with those found in healthy subjects (see **Supplementary Figure S5**). The top ten DEGs and top nine DEGs encoding secreted proteins were shown in **Figures 4D, E** respectively. To validate the above genomics data, we performed ELISA for TNFSF9, GDF15 and annexin A1 (ANXA1) as examples in the conditioned medium of rat BMMNC-derived macrophages. We found that the expressions of these genes were all upregulated in GDF15^high^ cells (**Supplementary Figure S6**). Functional clustering analysis on the DEGs revealed that GDF15^high^ macrophages were associated with a transcriptional signature featuring altered expressions of genes involved in cellular redox homeostasis and detoxification. These functional clusters included GO (Gene Ontology) terms cellular response to cadmium ion, cellular response to metal ion, detoxification of copper ion, cellular response to zinc ion, and cellular response to copper ion; UniprotKB Keywords cadmium, metal-thiolate cluster, and copper; InterPro classifications metallothionein domain, metallothionein superfamily (eukaryotic), and metallothionein (vertebrate, metal binding site) (all Benjamini *P* < 0.05). Since enrichment analyses do not provide a clear directionality, we therefore specifically examined upregulated and downregulated genes in relation to their functions as reported in the literature. GDF15^high^ macrophages exhibited upregulations in several genes with antioxidant/detoxification properties, including metallothioneins (MT1E/1G/1H/1M/1X/2A) (37) and ferredoxin reductase (FDXR) (38, 39). On the other hand, GDF15^high^ macrophages had downregulations in several genes with pro-oxidant properties, including thioredoxin interacting protein (TXNIP) (40) and the NADPH oxidase subunit cytochrome b-245 beta chain (CYBB, aka NOX2 or gp91phox) (41). These changes indicated a possible decrease in oxidative stress in GDF15^high^ macrophages. We also paid attention to genes which were known to be crucial for fundamental immune/inflammatory functions of the macrophage. Among the DEGs, there were downregulations in cathepsins (CTSB/D/Z) (42), macrophage scavenger receptor 1 (MSR1) (43), major histocompatibility complexes (HLA-DQB1 and HLA-DRB1) (44), Fc gamma receptor Ia (FCGR1A) (44), and Fc epsilon receptor Ig (FCER1G) (45). Moreover, we noticed that, in addition to GDF15, several genes with documented anti-inflammatory functions were upregulated, including annexin A1 (46), dual specificity phosphatase 14 (DUSP14) (47), and TNF alpha induced protein 6 (TNFAIP6) (48). These bioinformatics results together indicated that GDF15^high^ macrophage might have distinct functional properties as compared to the conventional macrophage.

Our initial UMAP analysis revealed that the GDF15^high^ sub-population (C5) comprised two clusters (number 6 and 9 in **Figure 4B**). Although the expression level of GDF15 in cluster 6 was lower than that in cluster 9 (see **Figure 4D**), cluster 6 appeared to express more GDF15 than the remaining ones. Moreover, **Supplementary Figure S4** confirmed that pooled cells from clusters 6 and 9 exhibited characteristic GDF15 expression. Attempting to uncover the interrelationship between the 2 clusters, we performed RNA velocity analysis (49). As shown in **Supplementary Figure S7**, cluster 6 and cluster 9 represented cells with diverging differentiation potentials; it was unlikely that these 2 clusters denoted two sequential differentiation status on the same differentiation route. Although the cells in cluster 6 and cluster 9 were not exactly of the same identity, because of our primary interest in GDF15 in the present study, it was rational to group these cells into a single sub-population.

To identify possible effector cells of the GDF15^high^ macrophage, we created a cell-cell communication network map using CellChat software, which utilized a mass action-based model to quantify the probability of cell-cell communications on the basis of known ligand-receptor pairs and the transcriptome profiles of sequenced cells (35). The input data sets were from the GDF15^high^ macrophage population and the total peripheral blood mononuclear cell pool. As shown in **Figure 4F**, the most significant target cell types included memory CD4^+^ T cell, non-conventional FCGR3A^+^ monocyte, and the GDF15^high^ macrophage *per se*. The ligand-receptor pairs potentially involved in reciprocal communications between GDF15^high^ macrophage and other cell types were shown in **Figures 4G, H**. These ligand-receptor pairs included signaling pathways with recognized anti-inflammatory functions, such as TGF-β signaling, colony stimulating factor 1 (CSF1) signaling, and annexin A1 signaling. It was noted that GDF15 signaling appeared to be implicated only in macrophage-macrophage interactions.

Finally, to understand whether there was possibly a special sub-population of GDF15-expressing monocytes which could give rise to mature GDF15^high^ macrophage, we utilized a public scRNA-seq database, which integrated 178,651 mononuclear phagocytes encompass dendritic cells, monocytes, and macrophages from 13 different tissues (50) (the interface available at https://macroverse.gustaveroussy.fr/2021_MoMac_VERSE), and found that there was not a discernable cluster of monocytes with enhanced expression of GDF15 (**Supplementary Figure S8**), suggesting that the GDF15^high^ sub-population was formed *de novo* during the process of monocyte to macrophage differentiation.

### 3.6 Inflammatory activation is dampened in *in vitro* derived GDF15^high^ macrophages

**Figure 5:**
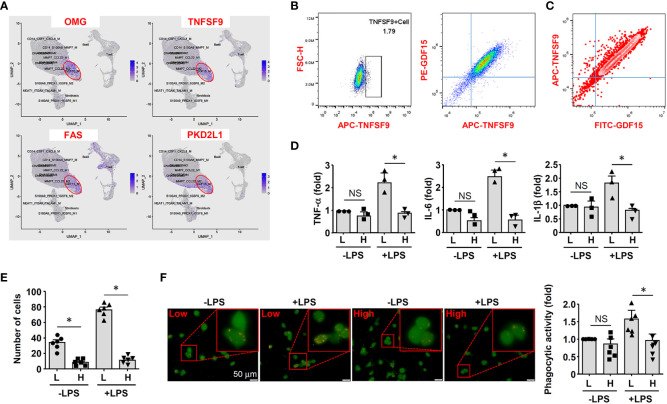
GDF15^high^ macrophages exhibited reduced inflammatory activation *in vitro*. **(A)** Expression patterns of potential substitute cell surface markers for GDF15 based on the scRNA-seq data. **(B)** Flow cytometry results showing that rat BMMNC-derived macrophages contained a minor fraction of TNFSF9^high^ cells, whose expression level was correlated with that of GDF15 (from 3 independent experiments). **(C)** Flow cytometry verification of the correlation between TNFSF9 and GDF15 expressions in human PBMNC-derived macrophages (from 2 independent experiments). **(D)** Real-time PCR results showing that GDF15^high^ macrophages (H) exhibited reduced expressions of TNF-α, IL-1β and IL-6 in response to LPS stimulation (1 μg/mL for 6 hr), as compared to GDF15^low^ cells (L). Rat BMMNC-derived macrophages were FACS purified using TNFSF9 as a substitute marker for GDF15, and primed with IFN-γ (10 ng/mL for 12 hr). **(E)** Boyden chamber cell migration assay showing that GDF15^high^ macrophages (H) exhibited reduced migratory activity as compared to GDF15^low^ cells (L) in the absence and presence of LPS stimulation. **(F)** Representative fluorescent microscopic images and quantitative data showing that GDF15^high^ macrophages exhibited reduced phagocytic activity in the presence of LPS stimulation as compared to GDF15^low^ cells. Phagocytosis was assessed by internalization of fluorochrome-labeled latex beads (orange color). The macrophages were from CD68pro-GFP rats. Data were mean ± SEM. * *P <* 0.05, one-way ANOVA. NS, no significance.

Because GDF15 is mainly expressed intracellularly, we searched substitute cell surface markers that were suitable for FACS purification. By reanalyzing DEGs in the GDF15^high^ population using the scRNA-seq data, we identified several potential marker genes including OMG (oligodendrocyte myelin glycoprotein), TNFSF9 (tumor necrosis factor superfamily member 9), FAS (Fas cell surface death receptor) and PKD2L1 (polycystin 2 like 1 transient receptor potential cation channel) (**Figure 5A**). Since TNFSF9 (also known as 4-1BB ligand and CD137 ligand) is a typical transmembrane glycoprotein readily expressed in macrophages (51), we examined whether TNFSF9 could be a substitute marker for GDF15 using flow cytometry. We verified that rat BMMNC-derived macrophages contained a minor fraction of TNFSF9^high^ cells, whose expression level was correlated with that of GDF15 (**Figure 5B**). We also confirmed the correlated expression patterns between TNFSF9 and GDF15 in human PBMNC-derived macrophages (**Figure 5C**). Using TNFSF9 as a substitute marker, we obtained GDF15^high^ and GDF15^low^ macrophages (rat BMMNC-derived), which were then primed with IFN-γ and further stimulated with LPS. We demonstrated that GDF15^high^ macrophages displayed dampened inflammatory reactions to LPS stimulation as compared to GDF15^low^ macrophages, as evidenced by the lowered expressions of TNF-α, IL-1β and IL-6 (**Figure 5D**), reduced migratory activity (**Figure 5E**), and reduced phagocytosis (**Figure 5F**). GDF15^high^ macrophages showed decreased cell migration even in the absence of LPS stimulation.

### 3.7 *In vitro* derived GDF15^high^ macrophages can produce anti-inflammatory effects via paracrine mechanisms

**Figure 6:**
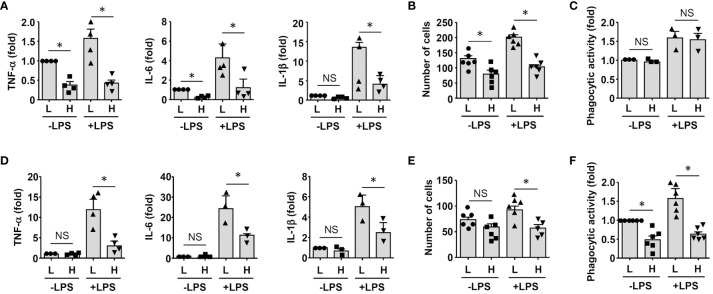
GDF15^high^ macrophages exerted anti-inflammatory effects via paracrine mechanisms. **(A-C)** RAW264.7 cells co-cultured with rat BMMNC-derived GDF15^high^ (H) or GDF15^low^ (L) macrophages were left untreated or stimulated with LPS for 4 hr. Results for the expression of pro-inflammatory cytokines (**A**, real-time PCR), cell migratory activity **(B)**, and phagocytic activity **(C)** were shown. **(D-F)** Unsorted rat BMMNC-derived macrophages co-cultured with rat GDF15^high^ (H) or GDF15^low^ (L) macrophages were left untreated or stimulated with LPS for 4 hr. Results for the expression of pro-inflammatory cytokines (**D**, real-time PCR), cell migratory activity **(E)**, and phagocytic activity **(F)** were shown. Data were mean ± SEM. * *P <* 0.05, one-way ANOVA. NS, no significance.

**Figure 7:**
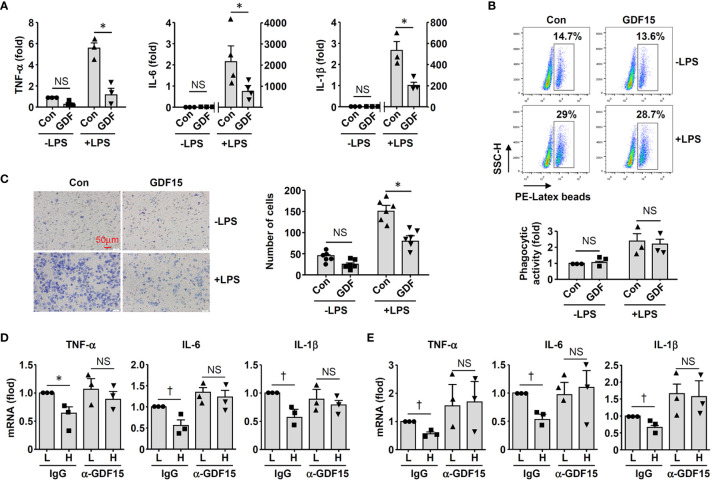
GDF15 might be a macrophage-derived anti-inflammatory factor. **(A)** Real-time PCR results showing that treatment with exogenous GDF15 (20 ng/mL) inhibited LPS-induced expression of pro-inflammatory cytokines in RAW264.7 cells. **(B)** Flow cytometry results showing that GDF15 treatment had no effects on phagocytosis in RAW264.7 cells without or with LPS stimulation. **(C)** Representative images and quantitative data of Boyden chamber assay showing that exogenous GDF15 inhibited migration of LPS-challenged RAW264.7 cells. Cells on the membrane were stained with Giemsa. **(D)** Effects of conditioned medium from GDF15^high^ macrophages (H), as compared to the medium from GDF15^low^ cells (L), on the expression of pro-inflammatory cytokines in RAW264.7 cells. All experiments were performed in the presence of LPS stimulation. α-GDF15, GDF15neutralizing antibody; IgG, non-specific immunoglobulin control. **(E)** The same experiments as those in D carried out in rat BMMNC-derived macrophages. Data were mean ± SEM. * *P <* 0.05, one-way ANOVA; † *P <* 0.05, unpaired *t*-test. NS, no significance.

To clarify whether GDF15^high^ macrophages could affect the functions of other macrophages in a pro-inflammatory environment, we co-cultured murine RAW264.7 cells with GDF15^high^ and GDF15^low^ macrophages (rat BMMNC-derived) separately for 48 hr, and then the RAW264.7 cells were further challenged with LPS. We demonstrated that RAW264.7 cells co-cultured with GDF15^high^ macrophages showed reduced cytokine expressions and reduced cell migration, while the phagocytic activity was not different (**Figures 6A-C**). The inhibitory effects of GDF15^high^ macrophages were also partly observed in unchallenged RAW264.7 cells. To further validate these results, we repeated the co-culture experiments by replacing the RAW264.7 cell line with primary macrophages (derived from rat BMMNCs). As shown in **Figures 6D–F**, similar anti-inflammatory effects of the GDF15^high^ macrophage were all observed in these experiments. Unlike RAW264.7, the phagocytic activities of primary macrophages were decreased in the GDF15^high^ co-culture (both in the presence and absence of LPS stimulation) (**Figure 6F**). In order to confirm that the decreases in inflammatory reactions in the GDF15^high^ co-culture were indeed a result of paracrine release of certain anti-inflammatory factor(s) from GDF15^high^ macrophages, we tested whether GDF15 itself could be the responsible paracrine factor as inspired by the previous studies (12, 52, 53). Firstly, we demonstrated that pretreatment with exogenous GDF15 mimicked the anti-inflammatory effects of GDF15^high^ macrophage in LPS-challenged RAW264.7 cells (including reduced cytokine expressions and reduced cell migration, but with no effect on phagocytosis) (**Figures 7A-C**). Secondly, in both RAW264.7 cells and primary BMMNC-derived macrophages, we showed that the conditioned-medium from GDF15^high^ macrophages exhibited similar anti-inflammatory effects as GDF15^high^ co-cultures (**Figures 7D, E**), while co-incubation with a neutralizing antibody of GDF15 diminished the anti-inflammatory effects of the conditioned-medium.

### 3.8 GDF15^high^ macrophages are present in multiple human tissues

**Figure 8:**
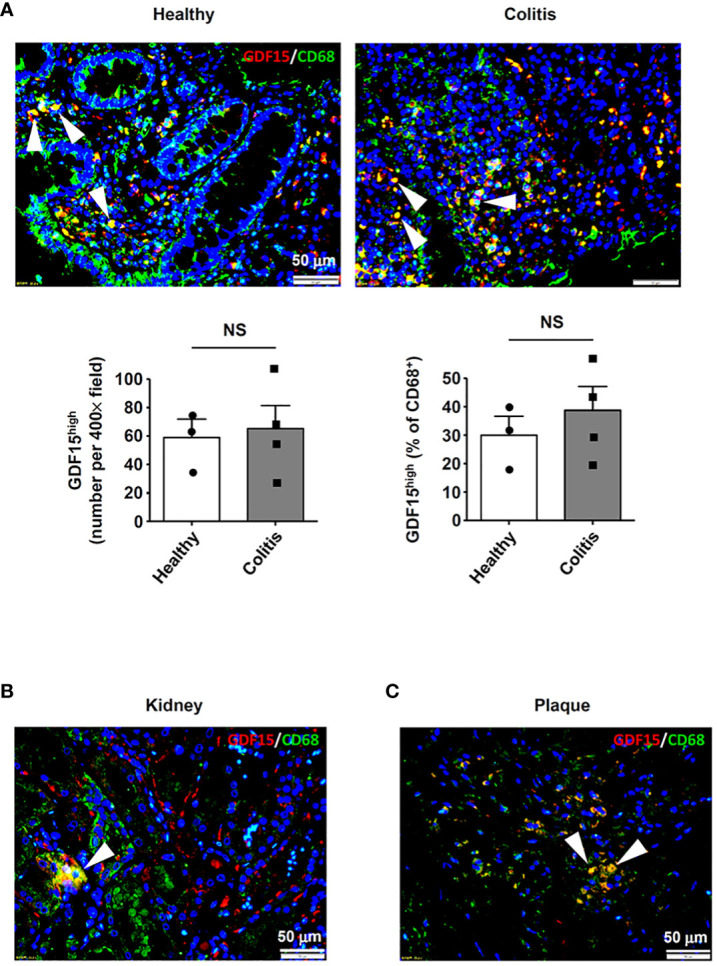
Detection of GDF15^high^ macrophages in various human tissues. GDF15^high^ macrophages (arrowheads) were identified using immunofluorescence double labeling with anti-CD68 (green color) and anti-GDF15 (red color) antibodies in **(A)** colon tissues from both healthy subjects and patients with ulcerative colitis, **(B)** kidneys (the normal peri-tumor tissue) (tested in one sample only) and **(C)** atherosclerotic plaques in the carotid artery (representative data from 6 independent samples showing similar results). The nuclei were counterstained with DAPI (blue). Data were mean ± SEM. NS, no significance (unpaired *t*-test).

In addition to the lungs, we also examined other human tissues which were known to contain abundant tissue-resident macrophages, including intestine (5) and kidney (3). In colon tissues from both healthy subjects and patients with ulcerative colitis, GDF15^high^ macrophages were detected in the lamina propria layer (**Figure 8A**). However, as compared to normal tissues, we failed to detect bulk macrophage infiltration in the colitis tissues as expected (**Figure 8A**); this pattern of macrophage distribution in inflammatory bowel disease was similar to the observations reported by Wright and colleagues (54). In parallel, there were no significant differences in the absolute or relative prevalence of GDF15^high^ macrophages between the healthy subjects and the patients with ulcerative colitis (**Figure 8A**). GDF15^high^ macrophages were also identified in kidney tissues (**Figure 8B**). Moreover, we found that GDF15^high^ macrophages were present in atherosclerotic plaques (**Figure 8C**), which were lesions profoundly composed of infiltrating macrophages in the sub-endothelial space (55). This result was consistent with previous studies (56). To confirm the co-expression of the signature marker genes in GDF15^high^ macrophages as identified by scRNA-seq, we performed GDF15/TNFSF9 immunofluorescence double labeling in human tissue sections. As shown in **Supplementary Figure S9**, cells with high expressions of both GDF15 and TNFSF9 could be identified in the lung, colon and kidney tissues. However, multicolor labeling in tissue sections with immunofluorescence for additional markers was technically unfeasible.

### 3.9 Rat lung macrophages contain GDF15^high^ clusters

**Figure 9:**
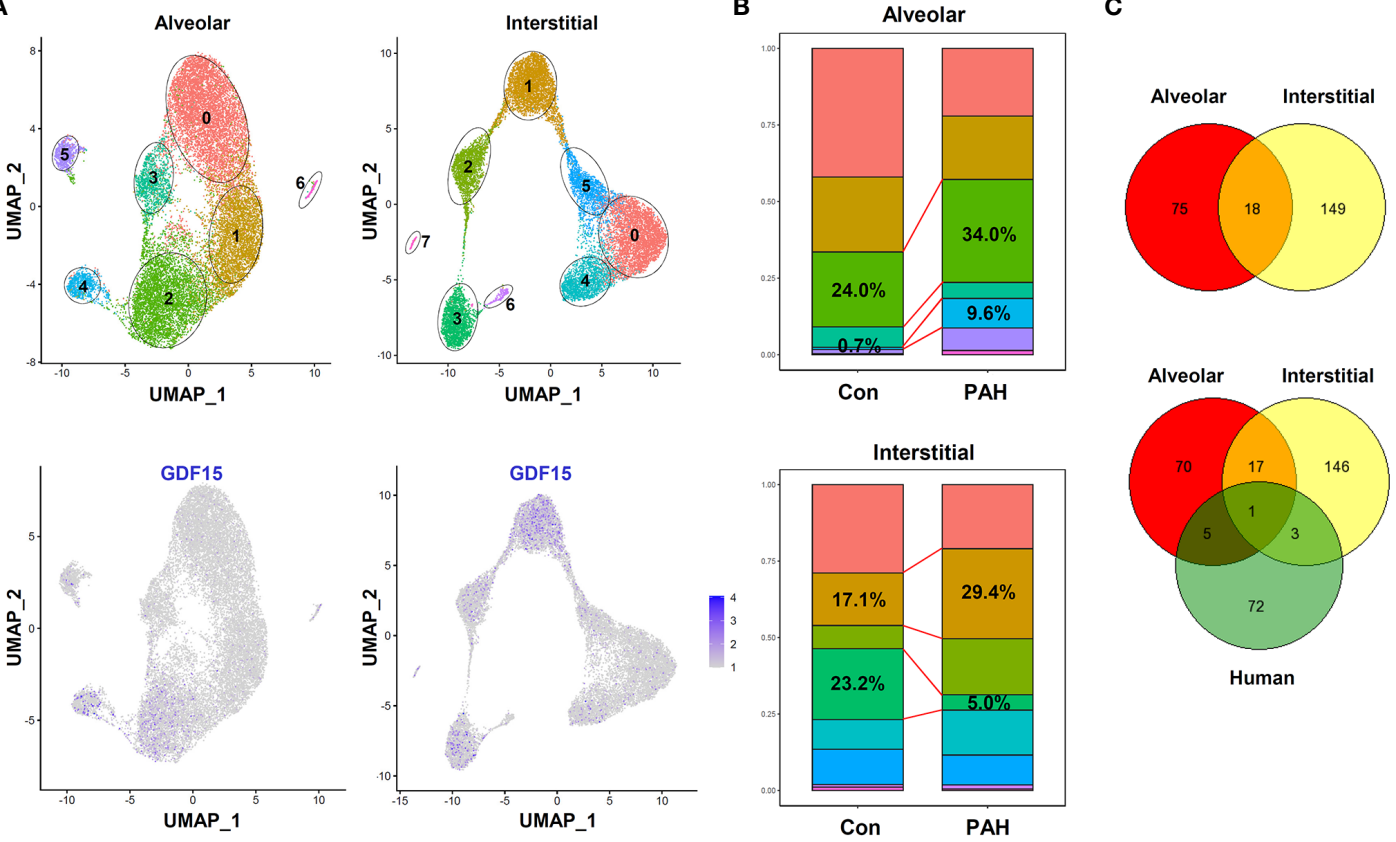
Results of scRNA-seq analysis showing the existence of GDF15^high^ clusters in rat lung macrophages. **(A)** Top, UMAP plots showing scRNA-seq clustering of freshly isolated lung alveolar macrophages and interstitial macrophages from rats (3 independent samples from Control group and 3 from experimental PAH group). Total 18977 alveolar macrophage cells (11842 from Control and 7135 from PAH) and 15357 interstitial macrophage cells (6768 from Control and 8589 from PAH) were analyzed. Bottom, patterns of GDF15 expression showing that clusters 2/4 in alveolar and clusters 1/3 in interstitial macrophages were GDF15^high^. **(B)** Comparison of the percentage prevalence of GDF15^high^ alveolar and interstitial macrophages between control and PAH animals. **(C)** Venn diagrams showing the numbers of differentially expressed genes between GDF15^high^ versus GDF15^low^ macrophages from rat lungs (Control group only) and from human PBMNCs.

To further confirm the presence of GDF15^high^ macrophages *in vivo*, we performed additional scRNA-seq experiments in freshly isolated lung macrophages from rats (3 independent samples from Control group and 3 from PAH group). The populations of alveolar macrophages and interstitial macrophages were distinguished using the algorism published previously (DOI: 10.18129/B9.bioc.SingleR) (57). We confirmed the existence of GDF15^high^ clusters in both alveolar macrophages and interstitial macrophages (clusters 2/4 and clusters 1/3 respectively in **Figure 9A**). The overall percentage prevalence of GDF15^high^ alveolar macrophage was increased in PAH (**Figure 9B**). Interestingly, we found that the GDF15^high^ alveolar macrophage and GDF15^high^ interstitial macrophage had largely distinct signature genes (**Figure 9C**). Likewise, human GDF15^high^ macrophage and rat lung GDF15^high^ macrophages shared few common signature genes (**Figure 9C**). We divided the lung macrophages into quartiles according to their GDF15 expression levels, and analyzed the DEGs between GDF15^high^ (top quartile) and GDF15^low^ (bottom quartile) cells (**Supplementary Table S5**). However, functional annotation analysis over the Gene Ontology and KEGG databases yielded paradoxical results, since in either alveolar or interstitial macrophages with or without PAH, the GDF15^high^ sub-population did not exhibit an anti-inflammatory phenotype. Instead, the expressions of IL-1α and various chemokines appeared to be higher in GDF15^high^ cells (see **Supplementary Table S5**).

## 4 Discussion

In the present study, we have verified the existence of a minor population of macrophages expressing a high level of GDF15 (GDF15^high^) in humans and animals, which have an inhibitory effect on the pro-inflammatory functions of other macrophages via a paracrine mechanism. Our findings have confirmed the previous histopathological observations showing the existence of GDF15^high^ macrophages in human lungs (22, 23). In addition, we have provided direct evidence that these GDF15^high^ macrophage cells are also present in other tissues/organs, at least under pathological conditions. Moreover, our results support the recent discovery of a population of monocyte-derived, GDF15-expressing macrophages in muscles, which have crucial roles in mediating tissue regeneration following inflammatory injuries (25). We have showed that the GDF15^high^ macrophage does not strictly belong to either the M1 or the M2 phenotype (2), highlighting the high degree of heterogeneity of macrophages which is more complex than that traditionally thought, as revealed by recent scRNA-seq studies (4, 24).

The developmental origin(s) of GDF15^high^ macrophages in the body is not clear. We have shown that GDF15^high^ macrophages can be derived from peripheral blood mononuclear cells by *in vitro* differentiation. Consistently, Patsalos et al. provided evidence suggesting that the GDF15-expressing regenerative macrophages found in injured muscles were infiltrating monocyte-derived in nature. Moreover, we have found that there are more GDF15^high^ macrophages in the lungs from experimental PAH models, where the inflammatory macrophage cells appear to mainly originate from blood-borne monocytes (58). Taken together, these observations suggest that GDF15^high^ macrophages may accrue locally as a consequence of monocyte infiltration at least during inflammatory insults. On the other hand, GDF15^high^ macrophages are also present in non-inflamed normal lungs, indicating that these cells are part of the tissue-resident macrophages. However, whether this GDF15^high^ population has an embryonic origin remains to be clarified.

Our results indicate that GDF15^high^ macrophages are associated with dampened responsiveness to pro-inflammatory activation; furthermore, they also inhibit the pro-inflammatory functions of other macrophages via a paracrine mechanism, and GDF15 *per se* appears to be a key mediator of the anti-inflammatory effects of GDF15^high^ macrophages. These findings are supported by the study of Patsalos et al., showing that the GDF15-expressing regenerative macrophages have anti-inflammatory properties (25). Specifically, GDF15 gene deletion resulted in an increase in the accumulation of F4/80^+^ macrophages in injured muscles, while exogenous GDF15 administration decreased the total number of infiltrating CD45^+^ myeloid cells (25). In Kupffer cells, Li et al. have demonstrated that GDF15 treatment attenuated LPS−induced expressions of inflammatory cytokines and inducible nitric oxide synthase (53). Interestingly, GDF15, being a paracrine anti-inflammatory factor, has also been observed in rat cardiac progenitor cells, of which allogeneic transplantation in the myocardium represses T cell-mediated inflammation and promotes M2 macrophage polarization (20). In addition, GDF-15 was also shown to inhibit the recruitment of polymorphonuclear cells following ischemic tissue injuries (18). Currently, the signaling mechanisms underlying the anti-inflammatory activity of GDF15 are not clearly understood. The (epi)genetic mechanisms governing the differentiation of GDF15^high^ macrophages remain elusive. Our present results suggest that GDF15^high^ macrophages are formed *de novo* during macrophage differentiation and/or maturation, but do not support the existence of a population of dedicated precursor cells (*i.e.* GDF15^high^ monocytes).

A limitation of the present study was that the functions of human GDF15^high^ macrophages were only characterized in cells derived in culture, while the functional phenotype of the tissue-resident GDF15^high^ macrophages remained unknown, because of the restricted access to adequate fresh human tissues suitable for cell isolation. To address this problem, we attempted using macrophage cells isolated from rat lung tissues with and without PAH, and performed additional scRNA-seq analysis. These results confirmed the presence of a GDF15^high^ sub-population in lung macrophages. However, our data also raised cautions for interpreting the nature of GDF15^high^ macrophages. Firstly, we found that the GDF15^high^ alveolar macrophage and GDF15^high^ interstitial macrophage had largely distinct signature genes, and human GDF15^high^ macrophage and rat lung GDF15^high^ macrophage shared few common signature genes too. These data were consistent with the notion that macrophages in the body display high degrees of heterogeneity and plasticity (4, 24, 59). In fact, neither the alveolar nor the interstitial macrophage population is homogeneous (60–63). Therefore, it is likely that GDF15^high^ macrophages in the body are not a uniform population depending on their origins, and it is impossible to use a set of common marker genes to define all GDF15^high^ macrophages. Secondly, we failed to show that the rat GDF15^high^ lung macrophages were associated with an obvious anti-inflammatory phenotype at the molecular level, although we argued that GDF15 *per se* released from these cells might potentially have paracrine anti-inflammatory effects. Moreover, we cannot exclude the possibility that the *in vitro* priming/induction protocol used in our study has a determinant role in inducing the anti-inflammatory phenotype in the resulting GDF15^high^ macrophage cells.

Indeed, the concept of “regulatory macrophage” was proposed more than a decade ago, based on the observations that some macrophages could produce a high level of IL-10 and exert inhibitory effects on immune responses (1). However, the identity of such a cluster of anti-inflammatory macrophages remains poorly defined because the current markers used to distinguish these cells are highly inconsistent and even controversial (64), and the borderline between regulatory macrophage and M2 macrophage is vague (65). The findings from the present study and other researchers (25) raise several interesting questions: (1) Can the functions of GDF15^high^ macrophage fulfill the requirement for a regulatory macrophage? (2) What are the specific surface markers that can be used to precisely define this macrophage sub-population? (3) What is the secretome profile of GDF15^high^ macrophage that is associated with its anti-inflammatory and tissue regenerative functions? Our study has confirmed that the distribution of GDF15^high^ macrophages is wide-spread in the body, at least under pathological conditions, indicating that these cells might be associated with some generic (rather than tissue-restricted) biological functions. In addition to the lungs, the presence of GDF15^high^ macrophages has also been reported in human atherosclerotic plaques (56). Moreover, our data suggest that during sterile inflammation, there is a trend of increase in the abundance of GDF15^high^ macrophages in the inflamed tissue, a process that might represent an intrinsic feedback mechanism to facilitate inflammation resolution and subsequent tissue repair. Nonetheless, the physiological and pathophysiological significance of the GDF15^high^ macrophage in human beings is to be further established by additional studies.

In summary, we have demonstrated that a minor population of peripheral and bone marrow mononuclear cells can be differentiated *in vitro* into macrophages that express a high level of GDF15. These GDF15^high^ macrophages do not exhibit a typical M1 or M2 phenotype but are associated with a unique molecular signature as revealed by single-cell RNA sequencing. Functionally, GDF15^high^ macrophages exhibit dampened responsiveness to pro-inflammatory activation and can also inhibit the inflammatory responses of other macrophages via a paracrine mechanism. GDF15 *per se* acts as at least partly the anti-inflammatory mediator released by GDF15^high^ macrophages. Moreover, GDF15^high^ macrophages are found to be present in multiple human tissues in the body. These data suggest that GDF15^high^ macrophages may represent a novel macrophage population with intrinsic anti-inflammatory functions, although their (patho)physiological importance remains to be explored.

## Data availability statement

The raw scRNA-seq dataset generated from human cells was deposited in the Genome Sequence Archive in National Genomics Data Center, China National Center for Bioinformation / Beijing Institute of Genomics, Chinese Academy of Sciences (Accession: PRJCA018194). The data are not made publicly available because of relevant government regulations. To request the raw data, please contact the corresponding author Dr. Xiaopei Cui via email (cuixiaopei@sdu.edu.cn). The scRNA-seq dataset from rat cells was deposited in the same repository which was publicly available (Accession: PRJCA023200).

## Ethics statement

The studies involving humans were approved by Human Ethics Committee of Shandong University Cheeloo College of Medicine. The studies were conducted in accordance with the local legislation and institutional requirements. The participants provided their written informed consent to participate in this study. The animal study was approved by Institutional Animal Ethics Committee of Shandong University Cheeloo College of Medicine. The study was conducted in accordance with the local legislation and institutional requirements.

## Author contributions

CD: Formal analysis, Methodology, Writing – original draft. HZ: Formal analysis, Methodology, Writing – original draft. ZZ: Formal analysis, Writing – original draft. CL: Writing – original draft. MM: Validation, Writing – original draft. HG: Writing – review & editing. QZ: Data curation, Funding acquisition, Investigation, Writing – original draft. XC: Conceptualization, Formal analysis, Funding acquisition, Methodology, Writing – original draft, Writing – review & editing. FJ: Conceptualization, Data curation, Formal analysis, Funding acquisition, Writing – original draft, Writing – review & editing.
