# Supplementary material for: Identification of a distinct cluster of GDF15high macrophages induced by in vitro differentiation exhibiting anti-inflammatory activities
Source: Front Immunol. 2024 Apr 8;15:1309739. doi: 10.3389/fimmu.2024.1309739 (PMC11036887; doi:10.3389/fimmu.2024.1309739)
Supplement: Supplementary file 1 [file DataSheet_1.pdf]

Supplementary Figure S1

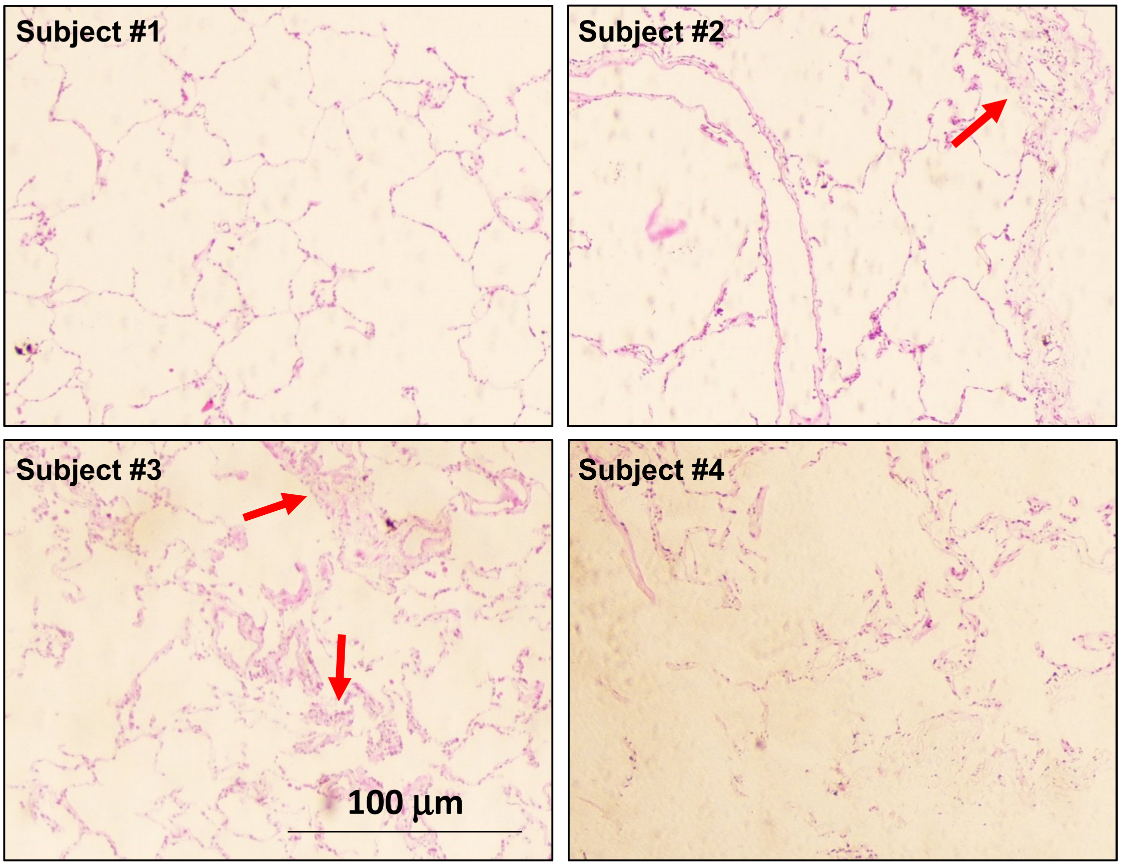

Figure S1. General histopathology of lung tissues from 4 individual patients with COPD. H&E stained sections showed that Subjects #1 and #4 had severe emphysema, resulting in poor cellularity of the lung tissue; in comparison, the lungs of Subjects #2 and #3 had significant inflammatory infiltrations as indicated by the patchy, densely cellularized areas (arrows).
