# Supplementary material for: Identification of a distinct cluster of GDF15high macrophages induced by in vitro differentiation exhibiting anti-inflammatory activities
Source: Front Immunol. 2024 Apr 8;15:1309739. doi: 10.3389/fimmu.2024.1309739 (PMC11036887; doi:10.3389/fimmu.2024.1309739)
Supplement: Supplementary file 2 [file DataSheet_2.pdf]

Supplementary Figure S2

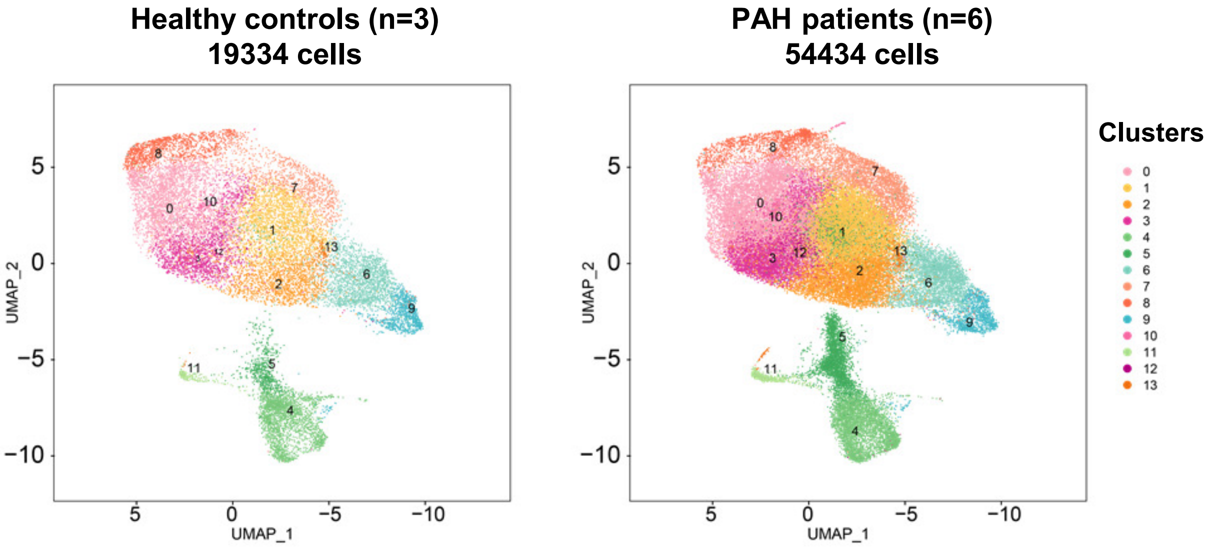

Figure S2. UMAP plots showing that macrophages from healthy controls and PAH patients had virtually identical clustering profiles when analyzed separately.
