# Supplementary material for: Identification of a distinct cluster of GDF15high macrophages induced by in vitro differentiation exhibiting anti-inflammatory activities
Source: Front Immunol. 2024 Apr 8;15:1309739. doi: 10.3389/fimmu.2024.1309739 (PMC11036887; doi:10.3389/fimmu.2024.1309739)
Supplement: Supplementary file 3 [file DataSheet_3.pdf]

Supplementary Figure S3

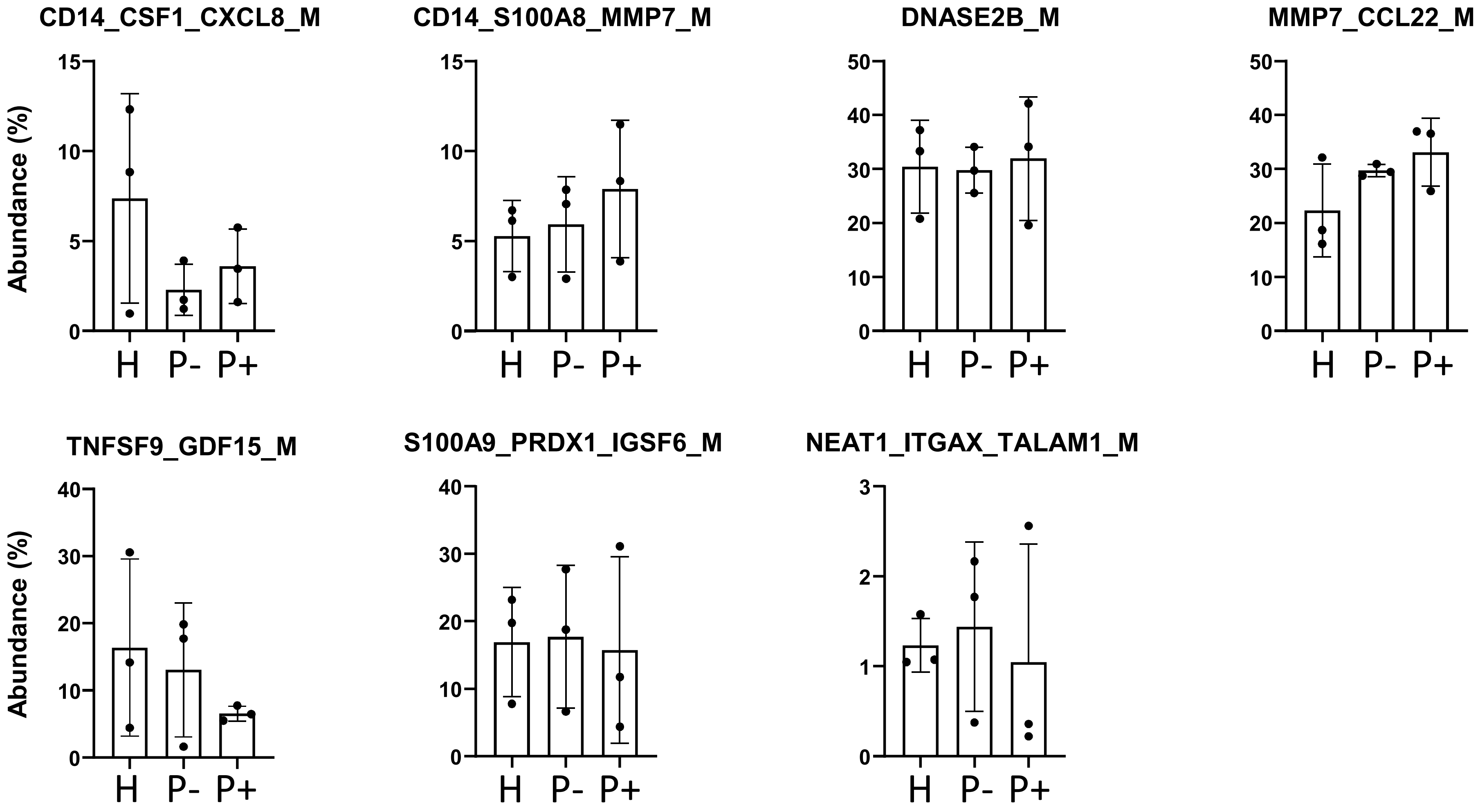

Figure S3. Quantitative data showing the percentage abundance of the 7 sub-populations of macrophages derived from human peripheral blood mononuclear cells. H, healthy subjects; P-, PAH patients without BMPR2 mutations; P+, PAH patients with BMPR2 mutations. Bars represent mean ± SD.
