# Supplementary material for: Identification of a distinct cluster of GDF15high macrophages induced by in vitro differentiation exhibiting anti-inflammatory activities
Source: Front Immunol. 2024 Apr 8;15:1309739. doi: 10.3389/fimmu.2024.1309739 (PMC11036887; doi:10.3389/fimmu.2024.1309739)
Supplement: Supplementary file 4 [file DataSheet_4.pdf]

Supplementary Figure S4

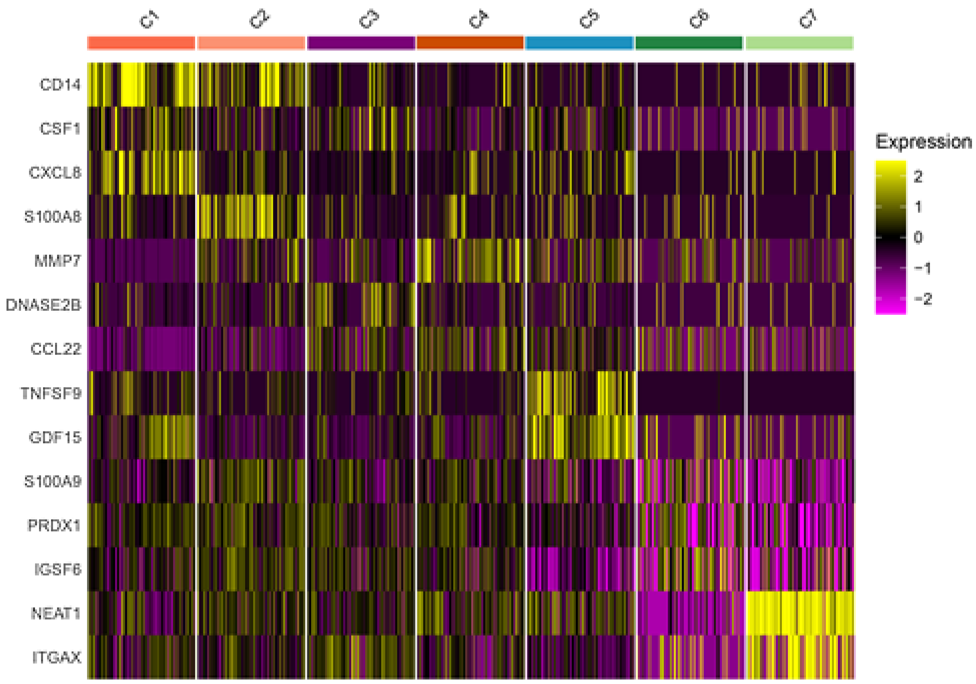

C1：CD14_CSF1_CXCL8
C2：CD14_S100A8_MMP7
C3：DNASE2B
C4：MMP7_CCL22
C5：TNFSF9_GDF15
C6：S100A9_PRDX1_IGSF6
C7：NEAT1_ITGAX

Figure S4. Heatmap plots showing the relative expression levels of the identified marker genes in different macrophage sub-populations (C1 to C7). The putative nomenclatures for these cell sub-populations were given below the graph.
