# Supplementary material for: Identification of a distinct cluster of GDF15high macrophages induced by in vitro differentiation exhibiting anti-inflammatory activities
Source: Front Immunol. 2024 Apr 8;15:1309739. doi: 10.3389/fimmu.2024.1309739 (PMC11036887; doi:10.3389/fimmu.2024.1309739)
Supplement: Supplementary file 5 [file DataSheet_5.pdf]

Supplementary Figure S5

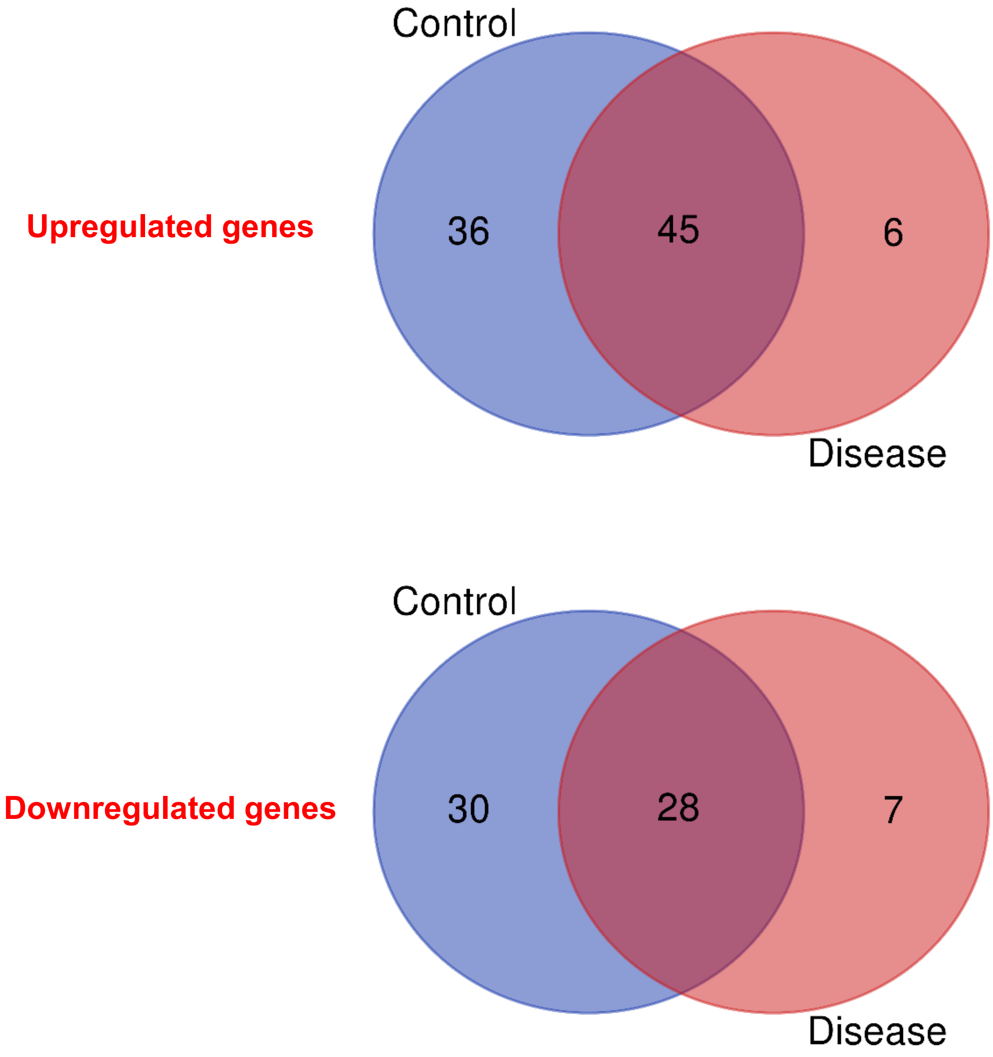

Figure S5. Venn diagrams showing the numbers of differentially expressed genes between GDF15^{high} and GDF15^{low} macrophages derived from 3 healthy subjects (Control) and 6 patients with pulmonary arterial hypertension (Disease), based on the scRNA-seq data set.
