# Supplementary material for: Identification of a distinct cluster of GDF15high macrophages induced by in vitro differentiation exhibiting anti-inflammatory activities
Source: Front Immunol. 2024 Apr 8;15:1309739. doi: 10.3389/fimmu.2024.1309739 (PMC11036887; doi:10.3389/fimmu.2024.1309739)
Supplement: Supplementary file 6 [file DataSheet_6.pdf]

Supplementary Figure S6

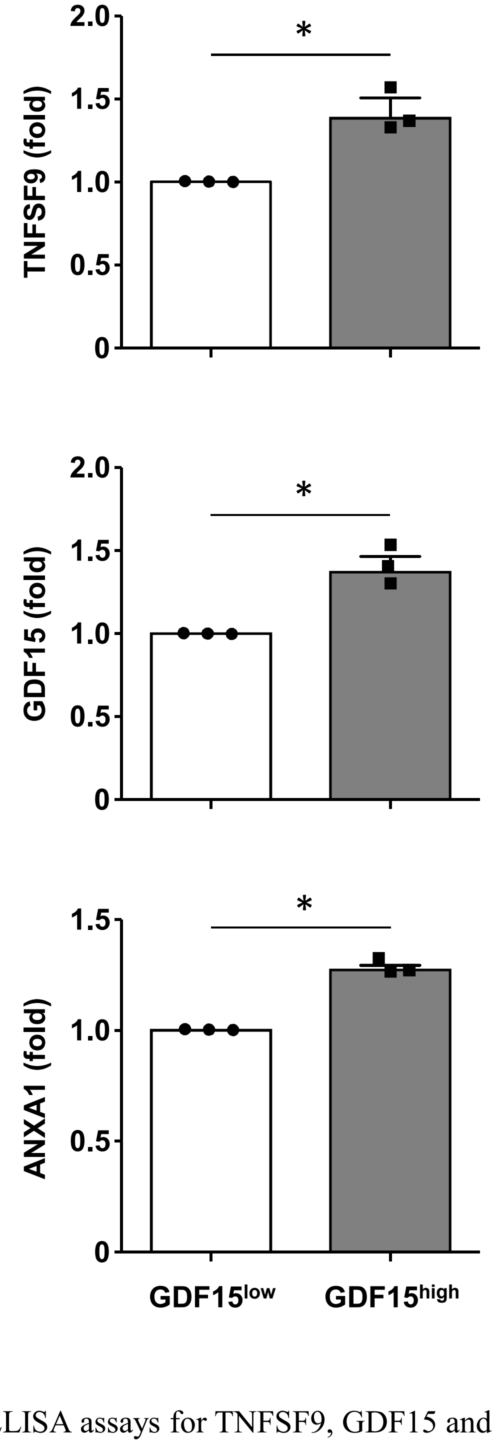

Figure S6. Results of ELISA assays for TNFSF9, GDF15 and annexin A1 (ANXA1) measured in the conditioned medium of rat BMMNC-derived macrophages. $GDF15^{low}$ and $GDF15^{high}$ cells were separated by FACS using TNFSF9 as a substitute marker. Data were expressed as mean ± SEM. * $P < 0.05$, unpaired *t*-test ($n = 3$ independent samples).
