# Supplementary material for: Identification of a distinct cluster of GDF15high macrophages induced by in vitro differentiation exhibiting anti-inflammatory activities
Source: Front Immunol. 2024 Apr 8;15:1309739. doi: 10.3389/fimmu.2024.1309739 (PMC11036887; doi:10.3389/fimmu.2024.1309739)
Supplement: Supplementary file 7 [file DataSheet_7.pdf]

Supplementary Figure S7

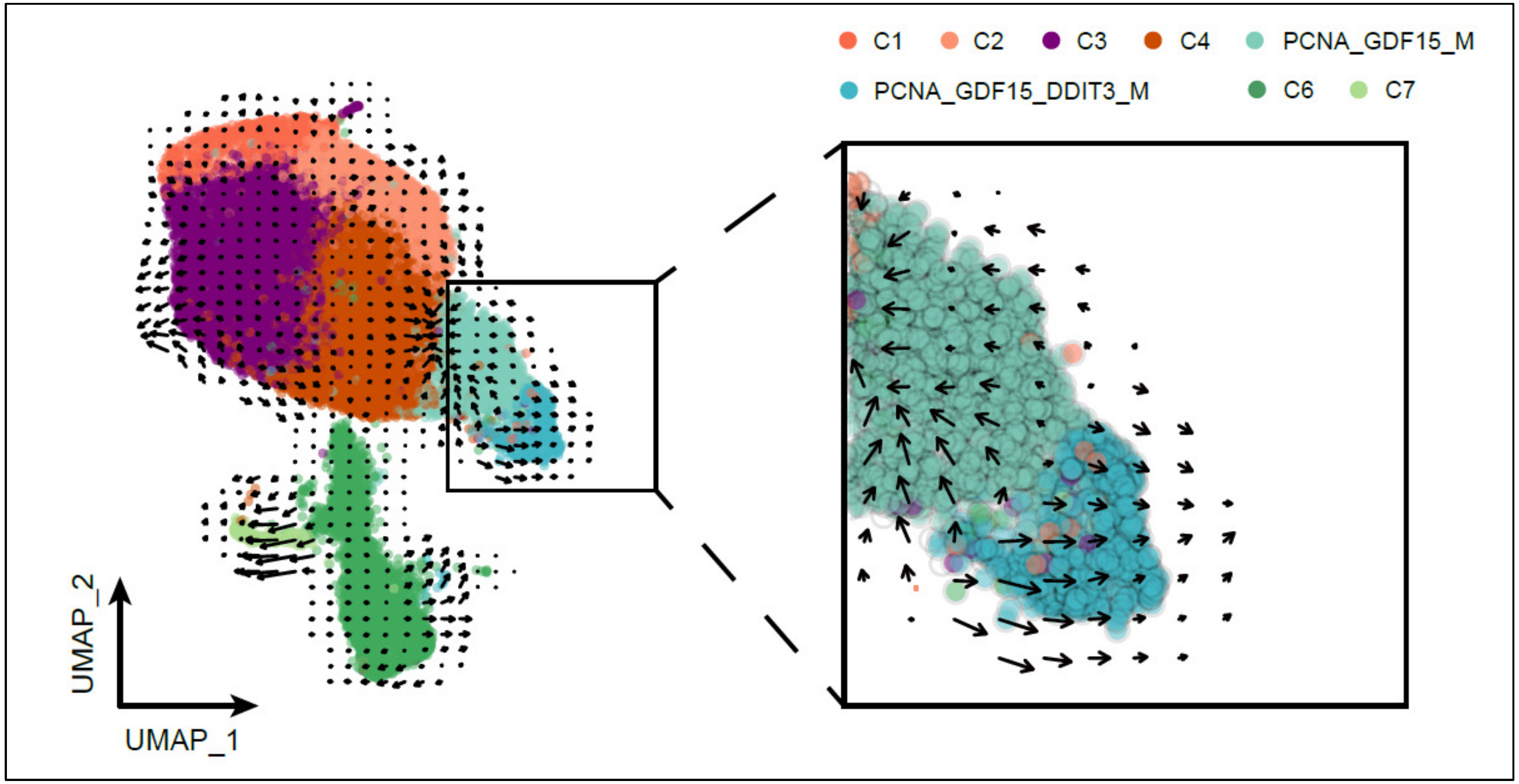

Figure S7. RNA velocity analysis result showing that the 2 sub-clusters of GDF15^{high} macrophage might represent cells of the same identity but with diverging differentiation potentials; it was unlikely that these sub-clusters denoted two sequential differentiation status on the same differentiation route.
