# Supplementary material for: Identification of a distinct cluster of GDF15high macrophages induced by in vitro differentiation exhibiting anti-inflammatory activities
Source: Front Immunol. 2024 Apr 8;15:1309739. doi: 10.3389/fimmu.2024.1309739 (PMC11036887; doi:10.3389/fimmu.2024.1309739)
Supplement: Supplementary file 8 [file DataSheet_8.pdf]

Supplementary Figure S8

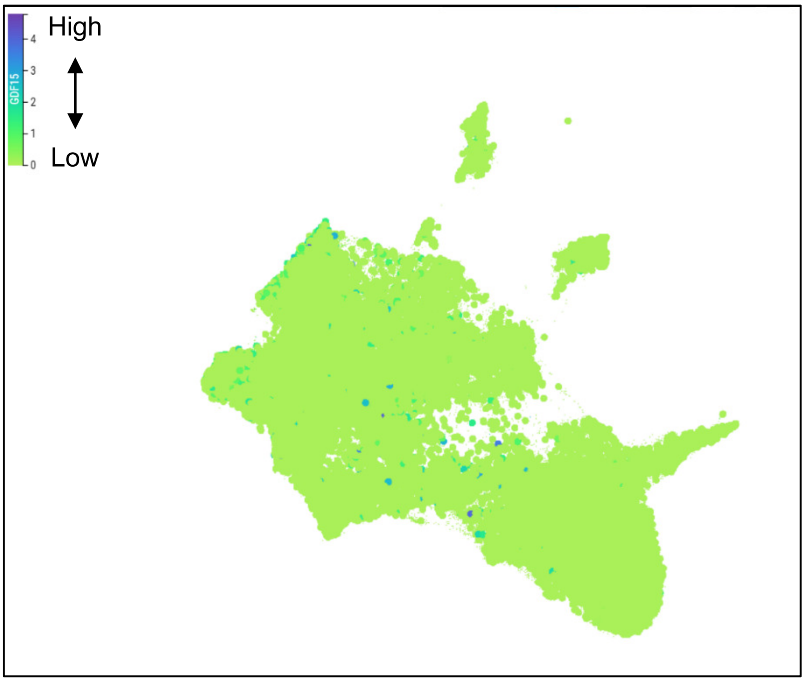

Figure S8. Analysis of the data in a public scRNA-seq database, MNP-VERSE, revealed that there was not a discernable cluster of monocytes with enhanced expression of GDF15. The color scale represented the relative expression level of GDF15.
