# Supplementary material for: Identification of a distinct cluster of GDF15high macrophages induced by in vitro differentiation exhibiting anti-inflammatory activities
Source: Front Immunol. 2024 Apr 8;15:1309739. doi: 10.3389/fimmu.2024.1309739 (PMC11036887; doi:10.3389/fimmu.2024.1309739)
Supplement: Supplementary file 9 [file DataSheet_9.pdf]

Supplementary Figure S9

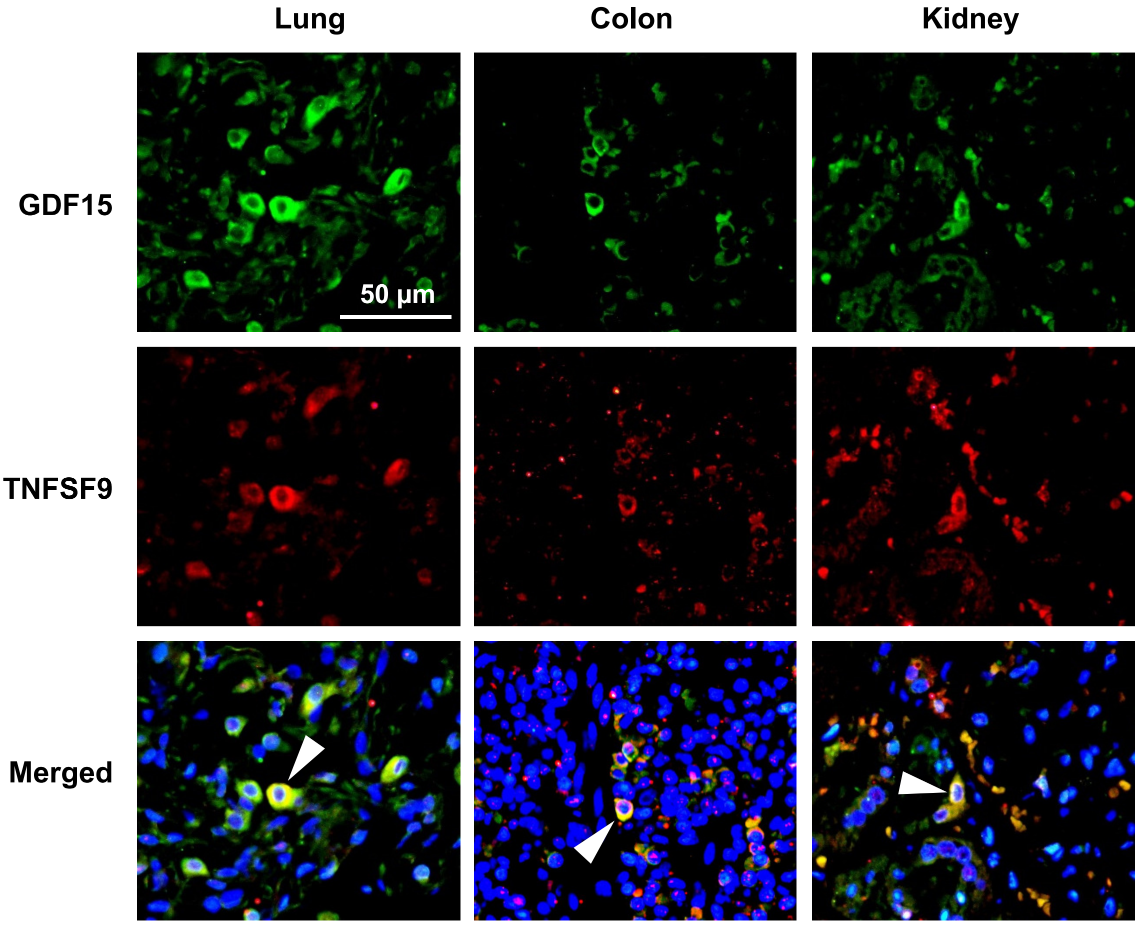

Figure S9. Images of immunofluorescence double labeling in human lung, colon (ulcerative colitis) and kidney tissue sections, showing the presence of cells with high expressions of both GDF15 and TNFSF9 (arrowheads).
