# Supplementary material for: Identification of a distinct cluster of GDF15high macrophages induced by in vitro differentiation exhibiting anti-inflammatory activities
Source: Front Immunol. 2024 Apr 8;15:1309739. doi: 10.3389/fimmu.2024.1309739 (PMC11036887; doi:10.3389/fimmu.2024.1309739)
Supplement: Supplementary file 10 [file Table_1.pdf]

Supplementary Table S1. Basic demographic data of human subjects

	Total number	Gender	Age	Disease condition
**Healthy volunteers**	7	All females	28-32	N/A
**PAH patients**	6	All females	28-32	Three patients had bone morphogenetic protein receptor type 2 mutations; all patients were on ambrisentan and tadalafil therapies
