# Supplementary material for: Identification of a distinct cluster of GDF15high macrophages induced by in vitro differentiation exhibiting anti-inflammatory activities
Source: Front Immunol. 2024 Apr 8;15:1309739. doi: 10.3389/fimmu.2024.1309739 (PMC11036887; doi:10.3389/fimmu.2024.1309739)
Supplement: Supplementary file 11 [file Table_2.pdf]

Supplementary Table S2. Sequences of the primers used for real-time PCR

Rat	***Forward***	***Reverse***
β-actin	CTCTGTGTGGATTGGTGGCT	CGCAGCTCAGTAACAGTCCG
IL-1β	GGGATGATGACGACCTGCTA	ACAGCACGAGGCATTTTTGT
TNF-α	ATGGGCTCCCTCTCATCAGT	GCTTGGTGGTTTGCTACGAC
IL-6	TTTCTCTCCGCAAGAGACTTCC	TGTGGGTGGTATCCTCTGTGA
Mouse	***Forward***	***Reverse***
β-actin	GGCTGTATTCCCCTCCATCG	CCAGTTGGTAACAATGCCATGT
IL-1β	GTGTCTTTCCCGTGGACCTT	AATGGGAACGTCACACACCA
TNF-α	CGGGCAGGTCTACTTTGGAG	ACCCTGAGCCATAATCCCCT
IL-6	CTTCTTGGGACTGATGCTGGT	CTCTGTGAAGTCTCCTCTCCG
