# Supplementary material for: Identification of a distinct cluster of GDF15high macrophages induced by in vitro differentiation exhibiting anti-inflammatory activities
Source: Front Immunol. 2024 Apr 8;15:1309739. doi: 10.3389/fimmu.2024.1309739 (PMC11036887; doi:10.3389/fimmu.2024.1309739)
Supplement: Supplementary file 12 [file Table_3.pdf]

Supplementary Table S3. Percentage frequencies of GDF15^{high} macrophages among the total population of CD68^{+} macrophages in both normal and COPD lungs

**Normal**	
Subject #1	20.7%
Subject #2	28.4%
Subject #3	11.4%

**COPD**	
Subject #1	20.9%
Subject #2	20.5%
Subject #3	16.1%
Subject #4	31.3%
